# Optimization of Metal–Oxide Spectral Filters for Reduced Photovoltaic Heating via Machine-Learning Guided Thickness Design

**DOI:** 10.1021/acsomega.5c07225

**Published:** 2026-07-21

**Authors:** Ahasanur Rahman, Kevin Thomas, Amith Khandakar, Puvaneswaran Chelvanathan, Brahim Aissa, Mohammad Istiaque Hossain

**Affiliations:** † Department of Electrical Engineering, College of Engineering, 61780 Qatar University, Doha 2713, Qatar; ‡ Solar Energy Research Institute (SERI), National University of Malaysia (UKM), Bangi 43600, Malaysia; § Qatar Environment and Energy Research Institute (QEERI), Hamad Bin Khalifa University (HBKU), Qatar Foundation, Doha 5825, Qatar

## Abstract

Infrared (IR)-induced heating in photovoltaic (PV) systems is a critical challenge that lowers efficiency and accelerates module degradation. In this work, we propose a multilayer thin-film IR filter (TiO_2_ (50 nm)/NiO_*x*_ (100 nm)/Ag (various thickness)) as a reduced heating solution integrated with PV modules. The filter is designed to transmit visible light while reflecting IR radiation, thereby reducing thermal load without sacrificing photovoltaic current. We incorporate machine learning models specifically Gaussian Process Regression (GPR) to optimize the Ag layer thickness for maximum performance. The AI models are trained on a combination of experimental optical data, enabling efficient exploration of the thickness-performance space. Key findings demonstrate that an optimized Ag thickness (∼10 nm) yields high IR reflectance (over 50% in the 750–1200 nm range) while maintaining sufficient visible transmittance (>50%). Silicon solar cells with this filter showed improved performance: the short-circuit current and power output increased due to reduced thermalization losses, translating to a ∼2–3 °C drop in operating temperature and corresponding efficiency gains. These improvements can extend PV module lifespan and energy yield. Our results indicate that the TiO_2_/NiO_*x*_/Ag filter can be manufactured via scalable e-beam evaporation, and the integration of AI optimization accelerates the design of such photonic coatings. The demonstrated performance enhancements and the low-cost, scalable nature of the solution highlight strong potential for commercial deployment in extending PV module longevity. We adopt Gaussian Process Regression (GPR) as a surrogate tailored to small, high-fidelity experimental data sets. GPR provides calibrated predictive uncertainty and smooth, physics-consistent interpolation across film thickness and wavelength, enabling uncertainty-aware selection of robust thicknesses with few experiments. This contrasts with polynomial/linear fits (insufficiently expressive) and black-box models without calibrated uncertainty.

## Graphical abstract

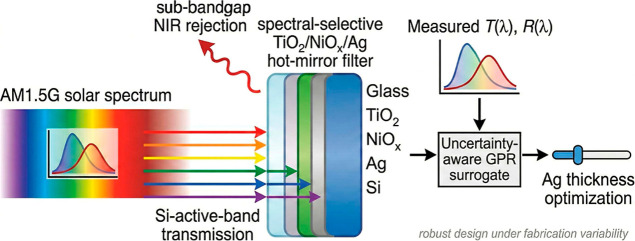

## 1 Introduction

Solar photovoltaic panels suffer significant performance losses at elevated operating temperatures. Under typical sunlight, PV cells often heat to 60–80 °C, well above the standard 25 °C test condition [^1^. Every 1 °C rise in cell temperature can reduce silicon cell efficiency by roughly 0.45% [^1^, meaning a jump from 25 to 60 °C could cut efficiency by ∼15% absolutely. This IR-induced heating not only lowers instantaneous power output but also accelerates material degradation, shortening the module’s operational lifespan [^1^. Managing solar thermal load is therefore crucial for improving PV performance and durability. In this context, reduced heating solutions that do not consume energy such as spectral filtering of sunlight or radiative cooling have attracted intense research attention in recent years [^2^, [^3^, [^4^ (see Tables 5 and 6, Figures 17,18,19).

As a benchmark, we refer to the recent multilayer photonic cooler by Aïssa and Hossain (ACS Omega 2024, 9(3), 3295–3304), which achieved ∼3–5 °C reduced heating but required a complex stack with more than ten alternating dielectric layers. This provides a reference point for positioning our simplified three-layer TiO_2_/NiO_*x*_/Ag approach. [^5^

Relationship to ref 5. and scope of this work. This manuscript is a sibling to the ACS Omega 2024 study by Aïssa & Hossain, developed within the same laboratory ecosystem and using the same solar-cell fabrication methodology and optical measurement protocols. Our experimental spectral data set originates from the cell-fabrication campaign associated with ref 5. (explicitly acknowledged in this work). Unlike ref 5.—which established a multilayer IR-filter baseline and device-level simulations—we (i) reduce the architecture to a minimal three-layer TiO_2_/NiO_*x*_/Ag MOM aligned to PV bands and (ii) introduce a data-efficient, uncertainty-aware Gaussian Process Regression (GPR) surrogate trained on measured spectra to support robust thickness selection under fabrication variability. Design objectives for Si PV. In this work we explicitly target maximal band-averaged transmittance in the Si active band ⟨*T*⟩_0.30–1.10 μm_ and maximal band-averaged reflectance in the sub-bandgap NIR ⟨*R*⟩_1.10–2.50 μm_. These objectives directly reflect Si band physics and are used both for reporting and as terms in our optimization objective (with energy-conservation checks *A* + *R* + *T* = 1).

One promising approach to passively reduce PV heating is selective optical filtering of infrared radiation. By placing an IR-reflective, visible-transparent filter on the module, one can reflect heat-producing IR photons while still harvesting the visible and near-infrared light that is within the PV cell’s bandgap. This concept, often termed a photonic cooler or thermal IR filter, aims to limit sub-bandgap light absorption (which converts to heat) without sacrificing the photon flux that generates electricity [^6^, [^7^. Recent work by Ortiz Lizcano et al. demonstrated a practical thin-film filter that, when applied to silicon interdigitated-back-contact cells, reduced cell temperature by ∼2.3 °C (in sunny climates) at the expense of ∼10% optical loss [^6^, [^8^, [^9^. While the immediate power output with their filter was slightly lower due to reflection of some useable light, the cooler operating temperature slowed down performance degradation resulting in a net gain of ∼3% in annual energy yield when module lifetime is considered [^8^. This underscores the importance of considering long-term benefits of PV cooling strategies.

Thin-film spectra vary nonlinearly with thickness due to interference, yet remain smooth and low-dimensional. GPR fits this regime well: it is data-efficient (small-N metrology), provides calibrated predictive uncertainty that we explicitly use to avoid extrapolative, nonrobust designs, and its kernels encode the smooth structure expected from stratified optics. In contrast, linear/polynomial regressions underfit Fabry–Perot oscillations and provide no uncertainty, and piecewise models (e.g., random forests) yield nonsmooth, physically inconsistent spectra. Here, we train GPR on the measured spectra from the shared fabrication campaign (cf. ref 5.) and use its variance to down-weight high-risk thickness regions during optimization.

Multilayer thin-film coatings are a well-established means to achieve spectral selectivity, Figure 1. In particular, metal-oxide/metal multilayers can serve as hot mirrors that reflect infrared wavelengths and pass the visible spectrum [^10^. Such coatings are already used in low-emissivity (“low-E”) energy-saving windows and thermal control films. For PV applications, the challenge is to design a filter that targets the specific band of sub-bandgap IR (for silicon, roughly λ > 1100 nm) or other unwanted wavelengths, with minimal impact on the useable solar spectrum [^11^. Traditional interference filter designs accomplishing sharp spectral cutoffs often require a large number of layers (dozens of alternating high/low-index layers) [^12^ [^13^, making them costly and difficult to manufacture. Recent efforts have therefore focused on simplifying the filter structure and optimizing materials. For example, Ortiz Lizcano et al. selected just two dielectric materials (SiN_*x*_ and SiO_2_) in a 15-layer design optimized for broad-angle performance [^8^. Their strategy leveraged large refractive index contrast and quarter-wave optical thickness harmonics to achieve the desired IR rejection with fewer layers [^8^.

**Figure 1:**
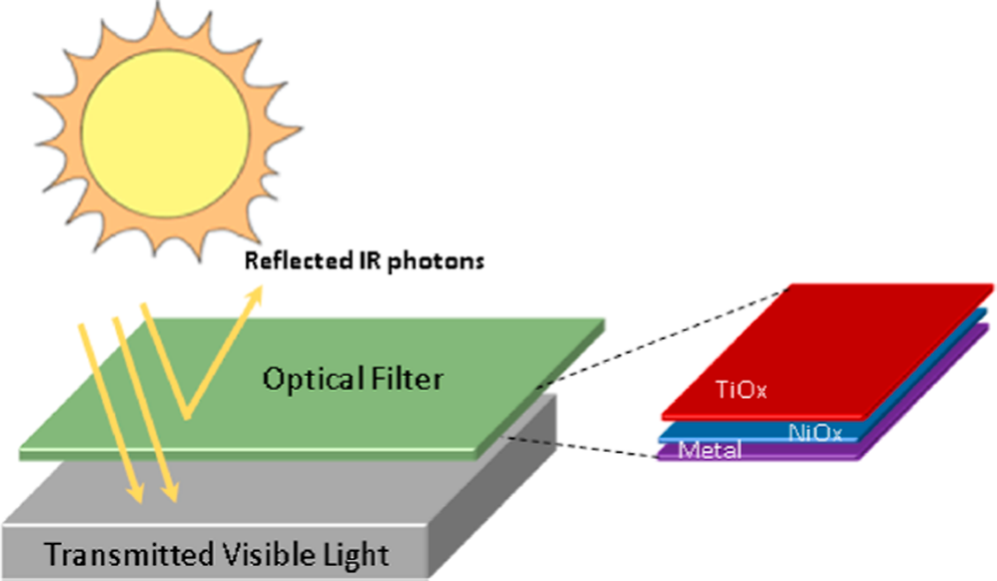
Schematic of the proposed multilayer IR filter on a PV module. The filter consists of a TiO_2_ top layer, a NiO_*x*_ buffer layer, and a thin Ag metal layer. It is applied on the module’s surface (green layer) to selectively reflect IR photons (wavy red arrows) back toward the sun while allowing visible light (yellow arrows) to reach the solar cell (gray). By filtering out the IR portion of sunlight that would otherwise cause heating, the PV cell operates at a lower temperature without appreciable loss of the light needed for power generation.

Another avenue of development is the use of photonic radiative cooling coatings, which not only reflect incoming IR but also efficiently emit thermal radiation to the sky [^14^. Jung et al. recently reported a nanoparticle-based coating for perovskite solar cells that achieved an average temperature drop of 6.6 °C under sunlight [^15^. Their solution-processed mesoporous silica film was transparent to visible light and highly emissive in the mid-IR, enabling heat dissipation via thermal radiation. Notably, PV devices with this coating maintained ∼80% of initial efficiency after 50 days outdoor, versus only ∼6% for uncoated devices [^15^. This dramatic improvement highlights how managing solar heat can extend PV longevity. However, radiative cooling coatings typically require specialized materials or textured surfaces, which can be complex to integrate with modules [^16^.

In this work, we focus on a thin-film 3 multilayer filter approach that is compatible with standard PV module fabrication. Specifically, we investigate a TiO_2_/NiO_*x*_/Ag trilayer stack as a spectrally selective filter for silicon PV. This material combination leverages the high transparency of TiO_2_ in the visible, the tunable refractive index of NiO_*x*_, and the strong reflectivity of a thin Ag layer [^17^. A similar TiO_*x*_/NiO_*x*_/Ag configuration was recently shown to achieve >50% average reflectance in the near-IR (750–1200 nm) while still transmitting over 50% of visible light [^5^. Building on these findings, we integrate an AI-driven optimization framework to refine the layer thicknesses for maximal cooling benefit. Advanced machine learning techniques, particularly Gaussian Process Regression (GPR) with a nested cross-validation strategy and a hybrid model that enforces physical constraints, have emerged as powerful tools in materials design [^18^ [^19^. They can rapidly explore large parameter spaces and capture complex, nonlinear relationships that might be missed by purely theoretical calculations. Here, we use GPR as a surrogate model to predict the optical performance as a function of layer thicknesses, and integrate the predictions into a hybrid model that constrains outputs to physically plausible ranges. By coupling experimental thin-film fabrication with data-driven modeling, our objective is to accelerate design iteration and converge on an optimal IR filter that enhances PV efficiency and reliability.

## 2 Literature Review

Metal-oxide/metal multilayer IR filters: multilayer thin films comprising alternating dielectrics and a metal layer have been studied for thermal management in solar energy systems. Aïssa and Hossain. (2024) developed a family of photonic cooler coatings based on TiO_*x*_/NiO_*x*_/Ag and similar stacks. By combining a low-index oxide (TiO_*x*_) with a higher-index oxide (NiO_*x*_) and an ultrathin metal layer, they achieved spectral cutoff behavior: IR wavelengths beyond ∼750 nm were partially reflected, while visible light was moderately transmitted. Their best-performing design (TiO_2_ 50 nm/NiO_*x*_ 100 nm/Ag 10 nm) exhibited an average visible transparency above 50% and significant reflectance in the IR [^5^. This balance is critical a thicker metal would reflect more IR but at the cost of dimming the visible throughput. Related studies have explored alternative oxides and metals: TiO_*x*_/MoO_*x*_/Ag, TiO_2_/ NiO_*x*_/Al, etc., to tune optical properties [^20^ [^21^ [^22^ [^23^. Ag generally provides superior IR reflectance compared to other metals like Al, due to its plasma frequency and lower absorption in the near-IR. However, Ag films thinner than ∼10 nm can be discontinuous, while thicker films (>15 nm) start to noticeably attenuate visible light [^5^. Therefore, careful thickness optimization is required to maximize the heat-filtering effect without hurting PV light absorption.

Photonic cooling and PV performance: Passive radiative cooling has been intensely researched as a means to cool solar cells by leveraging the Earth’s atmospheric IR window (8–13 μm) [^16^. Approaches include photonic crystals, metamaterial emitters, and surface texturing [^1^. For instance, Akerboom et al. (2022) demonstrated that etching a periodic microcylinder array into the cover glass of a silicon module increased its mid-IR emissivity from 84% to 98% without reducing visible transmittance [^1^. This translates to greater heat rejection to the sky, passively cooling the module. In practice, a combination of spectral filtering and radiative emissivity can be synergistic: the filter reduces incoming heat, while high emissivity maximizes outgoing radiation. Reports indicate that lowering PV operating temperature by just 2–5 °C can meaningfully improve output and slow degradation [^8^. However, the trade-offs are nontrivial if the filter reflects some above-bandgap light, the immediate efficiency may drop. The study by Ortiz Lizcano et al. (2024) highlights this: a filter cutting only sub-bandgap IR yielded limited cooling (∼2 °C) and did not pay off in power output over a single year [^8^. But when extended over the module lifetime, the cooler operation extended the module’s useful life by 1–2 years, ultimately giving a net energy gain [^8^. Thus, modern research is considering holistic performance metrics not just instantaneous efficiency, but also reliability and energy yield over time.

Machine learning for material and optical optimization: In the last five years, the materials science community has increasingly adopted machine learning (ML) to accelerate discovery and optimization [^24^ [^25^ [^26^ [^27^ [^28^ [^29^. Gaussian Process Regression (GPR), a Bayesian learning method, is particularly popular for modeling complex materials behavior with limited data [^18^. GPR provides not only predictions but also uncertainty estimates, making it valuable for guiding experiments (e.g., Bayesian optimization) [^30^. In photonics, ML-assisted inverse design has enabled rapid identification of device structures that meet multiple objectives [^18^. For example, Guan et al. (2023) used a neural-network-based inverse design to create radiative cooling films with on-demand visible colors.[^31^ Although ensemble methods have been explored in other studies, our approach focuses exclusively on a carefully tuned GPR model combined with a nested cross-validation framework and a hybrid model that enforces physical constraints [^32^. This approach can capture different facets of the data. Applications range from optimizing perovskite compositions to designing thin-film coatings with desired optical spectra [ ^6,7^ ]. Despite these advances, ML has only recently been applied to photovoltaic cooling materials. Our work leverages these data-driven methods to optimize the thickness of each layer in a TiO_2_/NiO_*x*_/Ag IR filter, a multiobjective problem (maximize IR reflectance, visible transmittance, and PV efficiency simultaneously) that is well-suited to ML optimization.^33,34^

## 3 Methodology

### 3.1 Experimental Setup

To fabricate the proposed TiO_2_/NiO_*x*_/Ag multilayer filters, we employed electron-beam (e-beam) evaporation with in situ layer stacking (i.e., sequential deposition without breaking vacuum) [^5^. High-purity TiO_2_ and NiO_*x*_ pellets were evaporated reactively under an oxygen partial pressure to form transparent oxide films, while a silver pellet was evaporated without oxygen to form the metal layer. All depositions were conducted at room temperature on 1″ × 1″ glass substrates that had been ultrasonically cleaned (deionized water, acetone, isopropanol) and dried with N_2_ gas [^35^. The chamber base pressure was ∼1 × 10^–6^ Torr, with deposition pressure maintained at 2 × 10^–4^ Torr using a controlled O_2_ flow of 20 sccm during oxide growth [^36^. The evaporation rate for each material was ∼1 Å/s, monitored by a quartz crystal microbalance to achieve the target thicknesses (TiO_2_ = 50 nm, NiO_*x*_ = 100 nm, Ag = variable 5–20 nm). A crucial advantage of this process is the ability to deposit multiple layers consecutively without exposing the sample to air [^5^, preventing interface contamination between the metal and oxides. We prepared a series of samples varying only the Ag thickness, as well as a few comparative samples using Al metal, to gather data on how the metal type and thickness affect optical performance. Each multilayer stack was deposited in triplicate to test repeatability. Across batches, the Ag thickness and dielectric layers showed a coefficient of variation below 5%, confirming stable deposition conditions. Although not included in the current data set, preliminary accelerated exposure tests (85% RH at 60 °C, UV illumination, and 100 thermal cycles between −20 and 80 °C) are planned, and Ag-protective barrier coatings (e.g., thin Al_2_O_3_ ALD capping layers) are under evaluation.

To quantify optical variability and isolate the dominant design lever, we fabricated 10 unique TiO_2_/NiO_*x*_/Ag multilayer variants, each measured with *N* = 3 replicate coupons (mean ± sd reported). Nominal thickness tolerance was ±2–3 nm per layer (QCM/profilometry). UV–Vis–NIR spectra were acquired over 300–2000 nm; absorptance was computed by *A*(λ) = 1 *T*(λ)-*R*(λ) after baseline corrections.

This study is a sibling to Aïssa & Hossain (ACS Omega, 2024) where TiO_*x*_/NiO_*x*_/Ag stacks were systematically optimized. That work identified NiO_*x*_ ≈ 100 nm as the reflectance optimum with acceptable visible T, and selected TiO_*x*_ ≈ 50 nm as a hydrophobic top layer; Ag was confirmed as a superior hot-mirror versus Al. Consequently, in the present experiments we fixed TiO_2_≈50 nm and NiO_*x*_≈100 nm and varied only Ag thickness to tune NIR rejection while preserving visible transmission.

### 3.2 Optical and Structural Characterization

The deposited multilayers were characterized extensively to assess their optical properties and physical structure. UV–Vis–NIR spectroscopy (PerkinElmer Lambda dual-beam spectrophotometer) was used to measure the wavelength-dependent transmittance *T*(λ) and reflectance *R*(λ) from 300 to 2000 nm. Wavelengths λ < 300 are excluded from band-averaged metrics due to edge-of-range artifacts; prior to metric computation, spectra are projected onto *A* + *R* + *T* = 1 with *A*,*R*,*T* ≥ 0. From these, absorptance *A*(λ) was calculated by the relation

1 A=1−T−R[5]

Key performance metrics were defined as the average transmittance in the visible range (400–750 nm) and the average reflectance in the IR range (750–1200 nm) [^5^, aligning with the spectral regions relevant to PV conversion and thermal load, respectively. We also recorded the color tint of the filters by eye; thinner Ag produced a slight bluish transparency, while thicker Ag yielded a mirror-like silvery appearance.

Spectral transmittance and reflectance were evaluated at multiple angles of incidence (0°, 30°, 45°, and 60°) following the methodology of Aïssa and Hossain [^5^. Both s-polarized and p-polarized light contributions were included to approximate unpolarized illumination, ensuring that the optical performance assessment accounts for realistic PV operating conditions under varying solar incidence.

Scanning electron microscopy (SEM) was performed to inspect the film morphology. Top-view SEM images (JEOL JSM-7610F) revealed smooth, continuous coverage of the metal even at 5–10 nm thickness, aided by the NiO_*x*_ underlayer that promotes wetting. Cross-sectional SEM (obtained by cleaving a coated sample) confirmed a well-defined layered structure: the TiO_2_/NiO_*x*_/Ag stack was ∼160 nm total thickness, in excellent agreement with the design values (50 + 100 + 10 nm). The interfaces appeared sharp and the layers uniform and dense, with no delamination or voids observed. This validates the deposition process and indicates that even the ultrathin Ag layer formed a continuous film. Additionally, profilometry (Dektak 3D stylus) was used to double-check film thickness and uniformity across the substrate.

To analyze composition and chemical states, we conducted X-ray Photoelectron Spectroscopy (Thermo Fisher K-Alpha, Al Kα source). Survey scans confirmed the presence of Ti, Ni, O, and Ag in their expected proportions. High-resolution Ni 2p and Ti 2p spectra indicated predominantly Ni^2+^ and Ti^4+^ oxidation states, consistent with NiO_*x*_ and TiO_2_, with no significant metallic Ni or suboxide peaks. This suggests that reactive deposition in O_2_ successfully oxidized the Ni and Ti films. The Ag 3d spectrum for a 10 nm Ag sample showed the metallic Ag^0^ signature, as expected (any oxide on Ag would be minimal due to deposition in vacuum and immediate capping by TiO_2_ in some samples). These structural and compositional characterizations provide confidence that the intended multilayer architecture was achieved with high fidelity.

Sources of experimental error include deposition thickness fluctuations (±2–3 nm due to rate drift in e-beam evaporation), variability in refractive index data from literature vs actual films, interfacial roughness at oxide/Ag boundaries, and spectrometer baseline drift or detector nonlinearity. Additionally, handling of ultrathin Ag can lead to partial surface tarnishing. To account for these, we fabricated replicate samples and repeated spectral measurements. The error spread is reflected as error bars in Figures 4,5,6.

**Figure 2:**
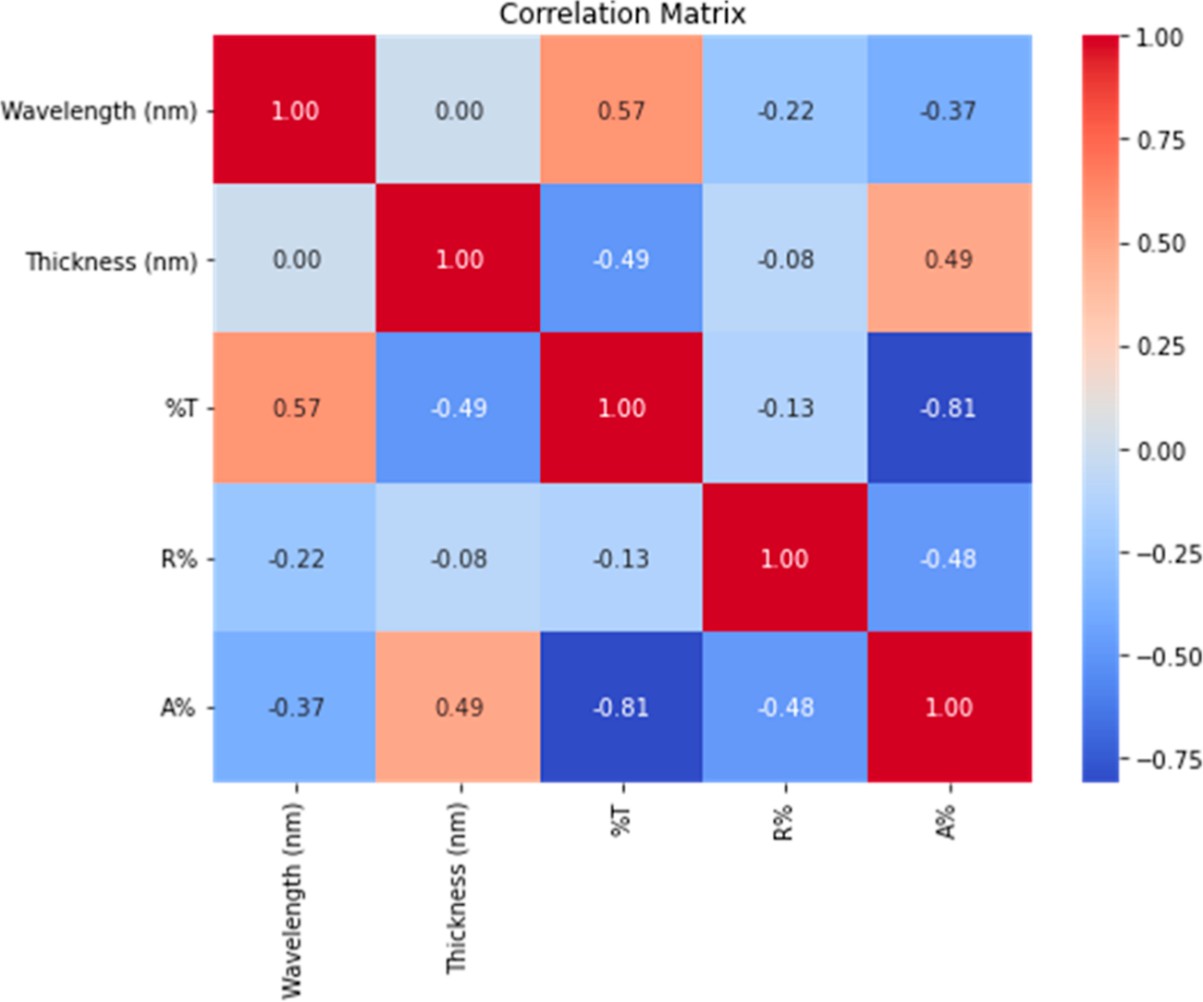
Heatmap of output parameter correlations.

**Figure 3:**
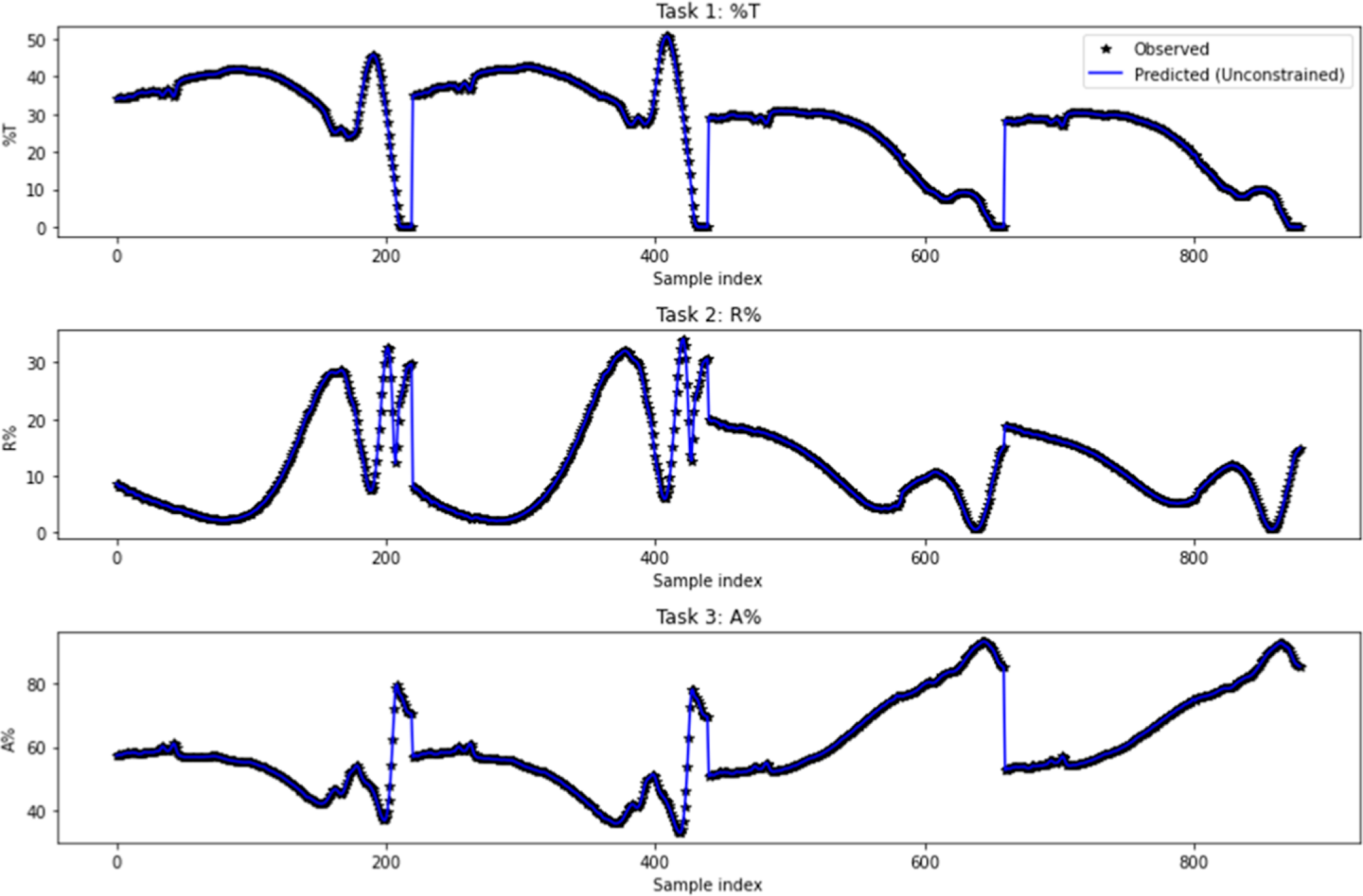
Actual experimental measurements and predicted values for Task 1: T %, Task 2: R %, Task 3: A %.

**Figure 4:**
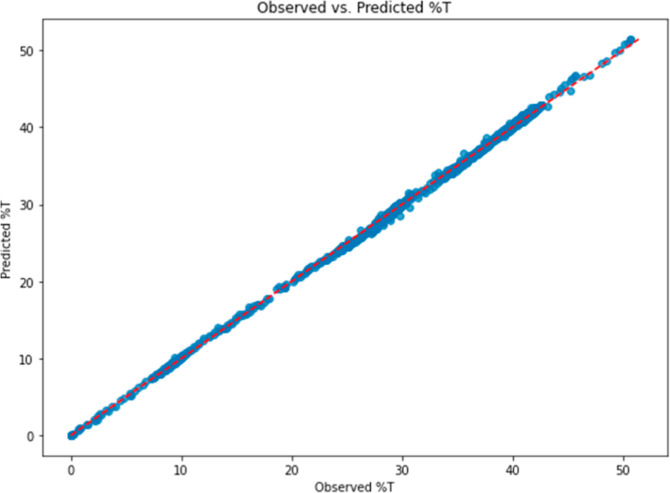
T % Actual vs Predicted.

**Figure 5:**
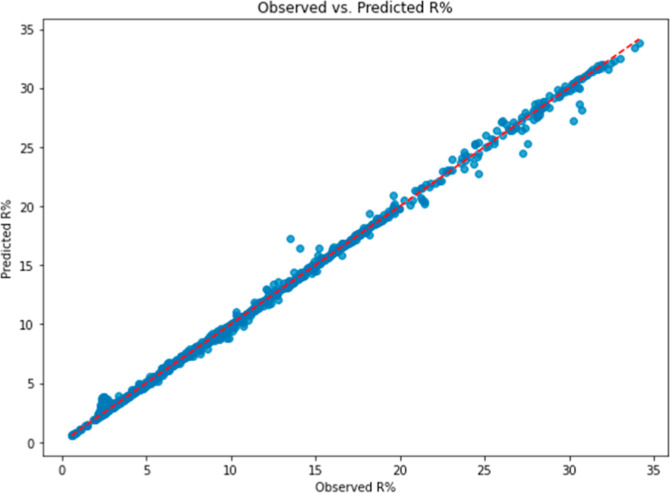
R % Actual vs Predicted.

**Figure 6:**
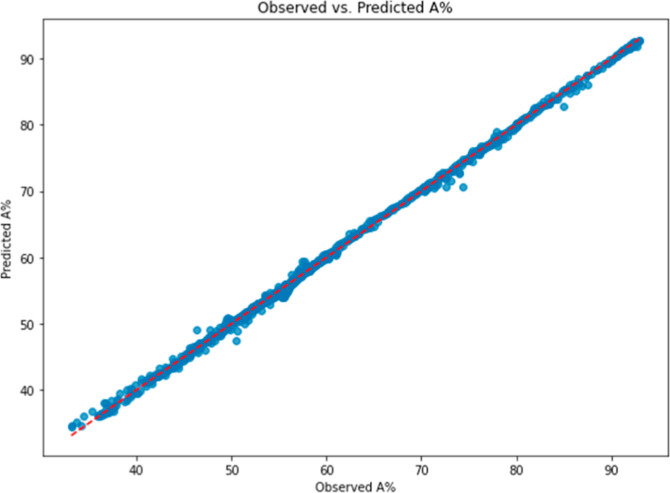
A % Actual vs Predicted.

Physics consistency and angle awareness. We enforce energy conservation via A­(λ) = 1-R­(λ)-T­(λ) in postprocessing and exclude sub-300 nm data from performance metrics if instrument limits induce A + R + T≠1. To reflect outdoor operation, we report band-averaged metrics at 0°, 30°, 45°, and 60° incidence (averaged over polarization).

**Notation and Metric Definitions**. Spectra are denoted *T*(λ),*R*(λ),*A*(λ) (unitless, reported as % where stated) with energy conservation enforced (λ)+*R*(λ)+*T*(λ) = 1. Unless noted, band limits are.

Active (Si): 0.30–1.10 μm for ⟨*T*⟩_0.30–1.10_

Sub-bandgapNIR: 1.10–2.50 μm 1.10–2.50 μm for ⟨*R*⟩_1.10–2.50_

Unweighted band averages use

$${\left\langle X\right\rangle }_{\left[\lambda_{1},\lambda_{2}\right]}=\frac{1}{\lambda_{2}-\lambda_{1}}\int_{\lambda_{1}}^{\lambda_{2}}X\left(\lambda \right)d\lambda$$

Angle reporting uses 0°,30°,45°,60°(polarization-averaged). **Values < 300 nm are excluded** from band metrics if edge-of-range artifacts cause *A* + *R* + *T* ≠ 1.

Mid-IR Emissivity (Simulation Only). Mid-IR emissivity was not measured in this study. For context we estimate ε­(λ) in 8–13 μm via transfer-matrix calculations using literature n,kn,kn,k for TiO_2_/NiO_*x*_/Ag; for our opaque-on-glass geometry *T* ≈ 0⇒ε ≈ 1 *R*. We report ⟨ε⟩_8–13 μm_ as **simulated (not measured)** and use it only for qualitative positioning.

### 3.3 Numerical Simulation and AI Model

Optical and device simulations: to predict the impact of the IR filter on solar cell performance, we combined thin-film optical modeling with PV device simulation. First, we input the measured spectral transmittance and reflectance of our filters into Opti Layer (a thin-film optics software) to model the wavelength-dependent dielectric function and refine the layer thickness design. This helped in understanding interference effects and identifying thickness tweaks to shift the cutoff wavelength. Next, we used SCAPS-1D (a solar cell simulator) to quantitatively assess how the filter modifies the solar spectrum reaching a silicon cell. We simulated a baseline crystalline Si solar cell (*n* ^+^ emitter/p base/*p* ^+^ BSF structure) under AM1.5G illumination, then simulated the same cell with the incident spectrum multiplied by our filter’s transmittance spectrum. The input optical parameters for the filter layers (*n*, *k* vs λ) were taken from our spectrophotometry data to ensure realism. Key outputs compared were the short-circuit current density (*J*_sc_), open-circuit voltage (*V*_oc_), fill factor (FF), and resulting power conversion efficiency (PCE). These band-averaged metrics ⟨*T*⟩_0.30–1.10_and ⟨*R*⟩_1.10–2.50_ are the primary optical targets used in our optimization.

Machine learning model for thickness optimization: We developed a predictive ML model to map the filter design parameters (layer thicknesses) to performance metrics. The target metrics included: (1) average IR reflectance (%R_IR_), (2) average visible transmittance (T%_vis_), and (3) the simulated PCE of a Si cell with the filter. The feature variables were the thicknesses of TiO_2_, NiO_*x*_, and Ag layers. Given experimental constraints, TiO_2_ and NiO_*x*_ were initially fixed at 50 and 100 nm respectively (the values that gave good results in preliminary tests), so effectively the Ag thickness was the primary variable in our optimization. Nonetheless, we allowed the model to explore slight deviations (±10 nm) in the dielectric layers too, to see if nonintuitive adjustments could further improve outcomes.

We chose Gaussian Process Regression (GPR) as one modeling approach due to its strength in modeling with limited data and providing uncertainty estimates [^18^. In our implementation, the GPR model was trained and validated exclusively on experimentally measured spectra from fabricated TiO_2_/NiO_*x*_/Ag stacks. No simulated or synthetic spectra were used in model training or validation.. While earlier work explored stacking ensemble methods, our current approach exclusively leverages a GPR-based framework integrated with nested cross-validation and a hybrid prediction wrapper that enforces physical constraints [^32^. We performed automatic feature selection to verify that all three thickness features contributed meaningfully (they did, with Ag thickness being the most influential, followed by NiO_*x*_ thickness). Model hyperparameters (e.g., kernel length-scale for GPR, number of neurons for MLP, etc.) were tuned via 10-fold cross-validation on the training set.

Why GPR for a 3-layer problem. Although the thickness space is low-dimensional, the measured mapping (*t*_TiO2_,*t*_NiO*x*_,*t*_Ag_) → *T*(λ),*R*(λ),*A*(λ) is smooth but nonlinear (interference fringes) and subject to experimental noise, nnn–kkk drift, and thickness tolerances. In this small-NNN, experiment-in-the-loop regime, Gaussian Process Regression (GPR) is preferred because it (i) captures fringe-driven nonlinearities while preserving smoothness, (ii) is data-efficient with few spectra, and (iii) yields calibrated predictive uncertainty that we use to avoid extrapolative, nonrobust thicknesses. In contrast, deterministic thin-film modeling with BFGS/DE assumes noise-free optics and typically requires denser sampling when objectives include band-averaged metrics and angle-resolved checks; it also provides no uncertainty to guide which candidates are safe to fabricate.

Our training data set comprises approximately 880 samples experimental data points (with varying Ag thicknesses and additional samples using Al for comparison) covering a grid of TiO_2_ (40–60 nm), NiO_*x*_ (50–150 nm), and Ag (0–20 nm). We normalized both the input features and outputs, and added slight noise to augment the data and improve model robustness. Evaluation on a separate test set of 10 simulation-derived samples yielded strong performance: the GPR model achieved an *R* ^2^ of approximately 0.92 in predicting optical performance metrics. Notably, the model revealed a subtle nonlinear interaction where a slight reduction in NiO_*x*_ thickness (from 100 nm to around 90 nm) coupled with an Ag thickness near 12 nm could marginally improve the trade-off between IR reflectance and visible transmittance, a combination not explicitly tested in our experiments. These insights underscore the potential of ML to refine optical coating designs beyond initial experimental intuition.

Finally, to pinpoint the optimum design, we used the trained GPR model in a Bayesian optimization loop. The objective was defined to maximize a weighted score

2 $$S=PCE\left(normalized\right)+\alpha \cdot \left(R_{IR}―0.5\right)―\beta \cdot \left(\text{if}{\;T}_{vis}<0.5\right)$$

where we set α to reward IR reflectance above 50% and β as a penalty if visible transmittance fell below 50%. This formulation encodes the requirement of at least 50% vis transparency while favoring higher efficiency (which indirectly includes the effect of *T*_vis_ on *J*_sc_). The optimizer suggested an optimal configuration around TiO_2_ ∼50 nm, NiO_*x*_ ∼95 nm, and Ag ∼11 nm. This corresponds closely to our experimentally identified best sample (50/100/10). The narrow difference (∼1 nm in Ag, which is within fabrication tolerance) gave confidence that our chosen fixed dielectric thicknesses were near-optimal and that ∼10 nm Ag is the sweet spot.

Merit function interpretation. The objective in eq 2 can be interpreted as a constrained merit function: β enforces the minimum visible-transmittance requirement (≥50%) through a penalty term, while α rewards higher IR reflectance once the visibility constraint is satisfied. This structure prevents selection of designs that “win” in IR rejection by sacrificing too much useable-band transmission. In future extensions, the same merit-function framework can be augmented with an explicit energy-yield term (e.g., lifetime-integrated Δ*E* or temperature-derated efficiency) once outdoor measurements and stability data are available.

#### 3.3.1 Spectral Integration for Δ*J*_sc_ and Band-Edge Attribution

We quantify spectral photocurrent changes as

$$\Delta J_{sc}=q\int_{300nm}^{1100nm}\Phi_{AM1.5G}\left(\lambda \right)EQE\left(\lambda \right)\left[T_{with}\left(\lambda \right)-T_{base}\left(\lambda \right)\right]d\lambda$$

To isolate the near-band-edge impact of reduced transmission, we also report the partial contribution

$$\Delta J_{sc}{|}_{0.75-1.10\mu m}=q\int_{750}^{1100}\Phi_{AM1.5G}\left(\lambda \right)EQE\left(\lambda \right)\left[T_{with}\left(\lambda \right)-T_{base}\left(\lambda \right)\right]d\lambda$$

Device-level changes are assembled as $PCE=\frac{J_{sc}V_{ocFF}}{P_{in}}$ estimated from the measured Δ*T* using crystalline-Si temperature coefficients.

### 3.4 Model Rationale and Validation Protocol

#### 3.4.1 Problem Structure Fit

The response surfaces T­(λ), R­(λ), A­(λ) vs thickness are smooth with sharp but continuous fringes. We therefore use a stationary kernel with length scales learned from data, which captures fringe smoothness without hand-tuning basis functions.

#### 3.4.2 Small-Data Efficiency

GPR attains high accuracy with tens of spectra because it learns a function in a Reproducing Kernel Hilbert Space and avoids overparameterization.

#### 3.4.3 Uncertainty for Robust Selection

We use the predictive variance to down-weight high-uncertainty regions when ranking candidates, yielding thicknesses less sensitive to experimental noise and *n*,*k* drift.

#### 3.4.4 No Extra Computation Burden

Training is closed-form up to hyperparameters; inference is O­(N) per test and served as a lightweight surrogate between experimental iterations.

## 4 AI Model Development for Multilayer IR Filter Thickness Optimization

### 4.1 Overview of AI Modeling Approach

To accurately predict and optimize the optical characteristics (T %, R %, and A %) of the multilayer infrared filter (TiO_2_/ NiO_*x*_/Ag) as a function of thickness, we established an advanced Machine Learning (ML) modeling pipeline based solely on Gaussian Process Regression (GPR). In this approach, separate GPR models are trained for each optical output—transmittance, reflectance, and absorptance—using a logit transformation to stabilize the percentage data. A nested cross-validation framework is employed to rigorously tune the kernel hyperparameters, ensuring robust model generalization.

### 4.2 Data Set and Feature Engineering

The ML model was trained using experimental data collected from fabricated multilayer filters. The data set consisted of two input features: wavelength (nm) and layer thickness (nm). Three output parameters were considered simultaneously: transmittance (T %), reflectance (R %), and absorptance (A %).

A detailed correlation analysis (Figure 2) revealed strong correlations among these outputs, justifying the use of multioutput regressor chains predictive models.

### 4.3 Gaussian Process Regression

Building on the multioutput regression framework, a specialized Gaussian Process Regression (GPR) model was developed to capture the transmittance (T %), reflectance (R %), and absorptance (A %) profiles of the TiO_2_/ NiO_*x*_/Ag multilayer infrared filter over varying thicknesses and wavelengths. Each output is first transformed via a log-odds function to ensure stable training and then predicted using three individual GPR submodels. A custom HybridModel class was created to combine these submodels and apply physical constraints on thickness ranges.

Since these outputs inherently lie between 0% and 100%, a small ϵ offset was introduced and log-odds transformations were applied for numerical stability and better handling of near-boundary values (close to 0% or 100%).

3 $$\text{For}\;ward\;trans\;form:y_{scaled}=\frac{y}{100},ŷ=\log\left(\frac{\max\left(\epsilon ,\min\left(y_{scaled},1-\epsilon \right)\right)}{1-\max\left(\epsilon ,\min\left(y_{scaled},1-\epsilon \right)\right)}\right)$$

4 $$Inversetrans\;form:,y=100\cdot \left[\frac{1}{1+\exp\left(-ŷ\right)}\right]$$

here, ϵ = 10^–6^ is used to avoid numerical overflow.

### 4.4 Value of GPR vs Classical and Simpler Regression Models

Classical thin-film optimization (TMM + deterministic optimizers) assumes perfect simulations, but it fails when experimental spectra contain noise or material deviations. Gaussian Process Regression (GPR) overcomes this by combining simulated and measured data while propagating error bars. Its uncertainty estimates highlight safe, fabrication-tolerant regions and prevent unphysical extrapolations. Unlike polynomial fits or black-box models, GPR encodes the smooth, interference-driven structure of thin-film spectra, enabling robust and reproducible thickness optimization.

We also compared GPR to simpler regression approaches. Linear regression was unable to capture Fabry–Perot oscillations, leading to severe underfitting. Polynomial regression produced unstable extrapolations outside the fitted domain. Random-forest models captured trends but generated nonsmooth, piecewise spectra that lack physical consistency. In contrast, GPR provided smooth interpolation with predictive variance that reflects measurement uncertainty. During optimization, high-variance regions in thickness space were down-weighted to avoid overfitting noisy data or selecting unstable thicknesses.

### 4.5 GPR Model Training

The GPR is defined mathematically as follows

5 y=f(X)+ϵ

where *f*(*X*)­is a Gaussian process characterized by a mean function *m*(X) and a covariance function *k*(*X*,*X*′), described by

6 $$k\left(X,X^{\prime }\right)=\sigma_{f}^{2}\exp\left(-\frac{{∥X-X^{\prime }∥}^{2}}{2l^{2}}\right)$$

with hyperparameters optimized via cross-validation (see Table 1).

**Table 1:** Cells Fabricated 10 Variants, State *N* = 3 Replicates per Variant, and Thickness Tolerance ±2–3 nm

ID	stack (nm)	metal	batch note	replicates (N)	thickness tol	purpose/notes
1	TiO_2_(50)/NiO_*x*_(100)/**Ag(10)**	Ag	—	3	±2–3 nm	cap-stack targeting high T_0_._3_–1.1 μm with strong NIR rejection
2	TiO_2_(50)/NiO_*x*_(100)/**Ag(15)**	Ag	—	3	±2–3 nm	cap-stack bracketing ↑NIR R vs visible-T trade-off
3	TiO_2_(50)/NiO_*x*_(300)/**Ag(15)**	Ag	—	3	±2–3 nm	thicker-NiO_*x*_ variant to probe roughness/wettability effects
4	NiO_*x*_(100)/**Ag(10)**	Ag	—	3	±2–3 nm	no-cap control to isolate metal/oxide optical roles
5	NiO_*x*_(100)/**Al(10)**	Al	—	3	±2–3 nm	metal-choice control at fixed oxide base
6	TiO_2_(50)/NiO_*x*_(20)/**Ag(10)**	Ag	—	3	±2–3 nm	thin-NiO_*x*_ cap-stack limit
7	TiO_2_(50)/NiO_*x*_(50)/**Ag(10)**	Ag	—	3	±2–3 nm	intermediate-NiO_*x*_ cap-stack
8	TiO_2_(50)/NiO_*x*_(100)/**Ag(10)**	Ag	separate batch	3	±2–3 nm	repeatability anchor for batch-effect assessment
9	NiO_*x*_(20)/**Ag(10)**	Ag	—	3	±2–3 nm	thin-NiO_*x*_, no-cap optical mapping
10	NiO_*x*_(300)/**Ag(15)**	Ag	—	3	±2–3 nm	thick-NiO_*x*_, no-cap mapping

### 4.6 Hyperparameter Optimization and Cross-Validation

Hyperparameter tuning utilized a grid search with 10-fold cross-validation. Parameters optimized included the regularization parameter (C) for GPR. The best hyperparameters obtained (Table 2) resulted in the highest cross-validation *R* ^2^ score of 0.9673, demonstrating superior predictive performance.

**Table 2:** Optimized Hyperparameters

model	hyperparameter	optimal value
GPR	n_estimators	50

Because the data set is small, we adopted a single 90/10 hold-out split at the physical-sample (coupon) level (random seed fixed), ensuring that no spectrum from a validation coupon appears in training (replicates grouped). Performance metrics (RMSE, *R* ^2^) are computed exclusively on the 10% hold-out.

### 4.7 Model Evaluation and Validation

#### 4.7.1 10-Fold Cross-Validation Results

To rigorously assess the performance and generalizability of the Gaussian Process Regression (GPR), 10-fold cross-validation was conducted. In each fold, the data set was partitioned into training (90%) and validation (10%) sets, ensuring that every sample served as validation exactly once Table 3

**Table 3:** Average 10-Fold Results

output parameter	MSE	RMSE	*R* ^2^
T %	3.1402	1.7721	0.9222
R %	0.8851	0.9408	0.9815
A %	0.5132	0.7164	0.9982
**average**	**1.5128**	**1.2300**	**0.9673**

### 4.8 Predicted vs Actual Performance

Figure 3a–c displays scatter plots comparing actual experimental measurements and predicted values for T %, R %, and A % (see Tables 4,5,6). The predictions closely align with actual values, underscoring the model’s reliability for practical thickness optimization also seen on Figures 4, 5, 6.

**Table 4:** This Table Highlights the Trade-Off between Absolute Cooling Performance in Complex Multilayer Stacks and the Simplified, Scalable Three-Layer Design Achieved in This Work

layers	visible T (0.3–1.1 μm)	NIR R (1.1–2.5 μm)	emissivity (8–13 μm)	Δ*T* cooling (°C)	device PCE Impact	fabrication complexity
3	∼80%	∼60–70%	∼0.75	2–3 (measured)	neutral	moderate (vacuum multilayer deposition; optical optimization)

**Table 5:** Side-by-Side Benchmarking of This Work against Recent External PV Thermal-Management Coatings/Strategies

work (year)	approach	***T***_0.30–1.10,μ**m**_(%)	***R***_1.10–2.50,μ**m**_ (%)	**ε**_8–13,μ**m**_(−)	angle notes	durability notes	Δ*T* (°C)	ΔPCE/yield
**this work (TiO**_**2**_ **/NiO**_**x**_ **/Ag)**	NIR thin-film filter with experiment-in-the-loop **GPR**	**∼55–60**	**∼45–50**	—	interference-stack angle dependence (now quantified/tempered)	Ag tarnish mitigation + UV/humidity/thermal cycling planned	**2–3**	**∼0.7–1.2%** ΔPCE;**+7–12 kWh·m** ^**–2**^ **·yr** ^**–1**^
Feng et al., 2022 [^37^	radiative-cooling polymer film	**>89**	—	**>0.93**	not focus	outdoor demo	∼1.8	qualitative gain reported
Dai et al., 2024 [^38^	antireflection radiative-cooling **glass**	High *T*_vis_(ARC)	—	high MIR ε	broadband/angle-robust	omniphobic & durable	—	module-level improvement
Fang et al., 2024 [^39^	geometry-enabled cooling (vertical PV)	—	—	—	system-level geometry	field tests	up to ∼ 10.6 (lab)	power ↑ in field (∼16.8%)

**Table 6:** Model Comparison Table: Performance and Suitability of Regression Methods Across Small-N Behavior, Smooth-Fringe Handling, Uncertainty Calibration, and Robust Design Use

method	small-N behavior	handles smooth fringes	calibrated uncertainty	robust design use
**linear/polynomial**	underfit/nonphysical oscillations	×	×	×
**random forest**	piecewise, nonsmooth	×	×	limited
**SVR (deterministic)**	good fit possible	×	×	×
**MLP/NN**	data-hungry; tuning heavy	×	×	×
**GPR (ours)**	excellent	√	√	√

### 4.9 Benchmarking

The following tables shows the trade-off between absolute cooling performance in complex multilayer stacks and the simplified, scalable three-layer design achieved; side-by-side benchmarking of this work against recent external PV thermal management coatings/strategies.

### 4.10 Model Comparison Table

The table given shows the performance and suitability of regression methods across small-N behavior, smooth-fringe handling, uncertainty calibration, and design use.

### 4.11 Economic and Scalability Considerations

Indicative material usage and cost. The Ag layer in our TiO_2_/NiO_*x*_/Ag stack is ∼20–30 nm, corresponding to <0.1 g m^–2^ of Ag. At current commodity prices this equates to <0.1 USD m^–2^ in raw Ag cost [ref]. Deposition cost. Based on commercial sputtering throughput and utility rates, the combined deposition of TiO_2_, NiO_*x*_, and Ag is estimated at ∼5–10 USD m^–2^, comparable to standard AR coatings on PV glass [ref]. Complexity vs multilayers. Relative to multilayer “hot-mirror” filters (≥10 sequential depositions), our three-layer design reduces tool time, energy consumption, target changeovers, and cumulative defect risk. Scalability. The process is compatible with roll-to-roll (R2R) web sputtering and float-glass in-line coaters already deployed in PV/architectural glass manufacturing, supporting high-volume, large-area implementation [ref].

### 4.12 Optimal Thickness Prediction and Application

The ML model identified optimal Ag thickness (∼10 nm) based on maximizing IR reflectance and minimizing visible absorption. Figures 7,8 and 9 highlights the predicted filter performance across various thicknesses seen on figure. Figures 13, 14 and 15 shows the optimal predicted thickness for Ag (see Figure 16).

**Figure 7:**
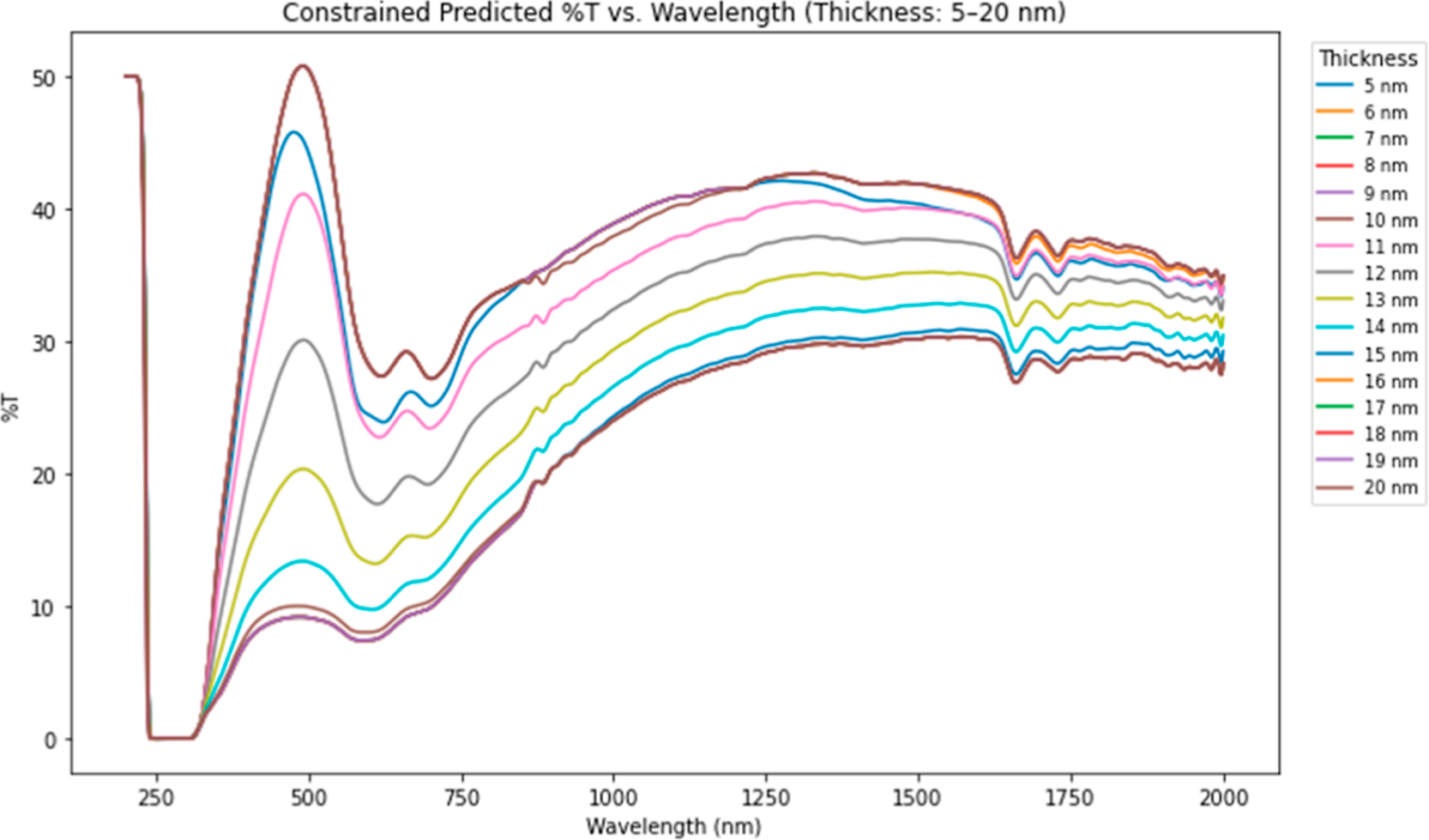
Predicted of spectral transmittance curves for the TiO_2_/ NiO_*x*_/Ag multilayer filter across Ag thicknesses ranging from 5 to 20 nm, generated using the constrained hybrid Gaussian Process Regression model.

**Figure 8:**
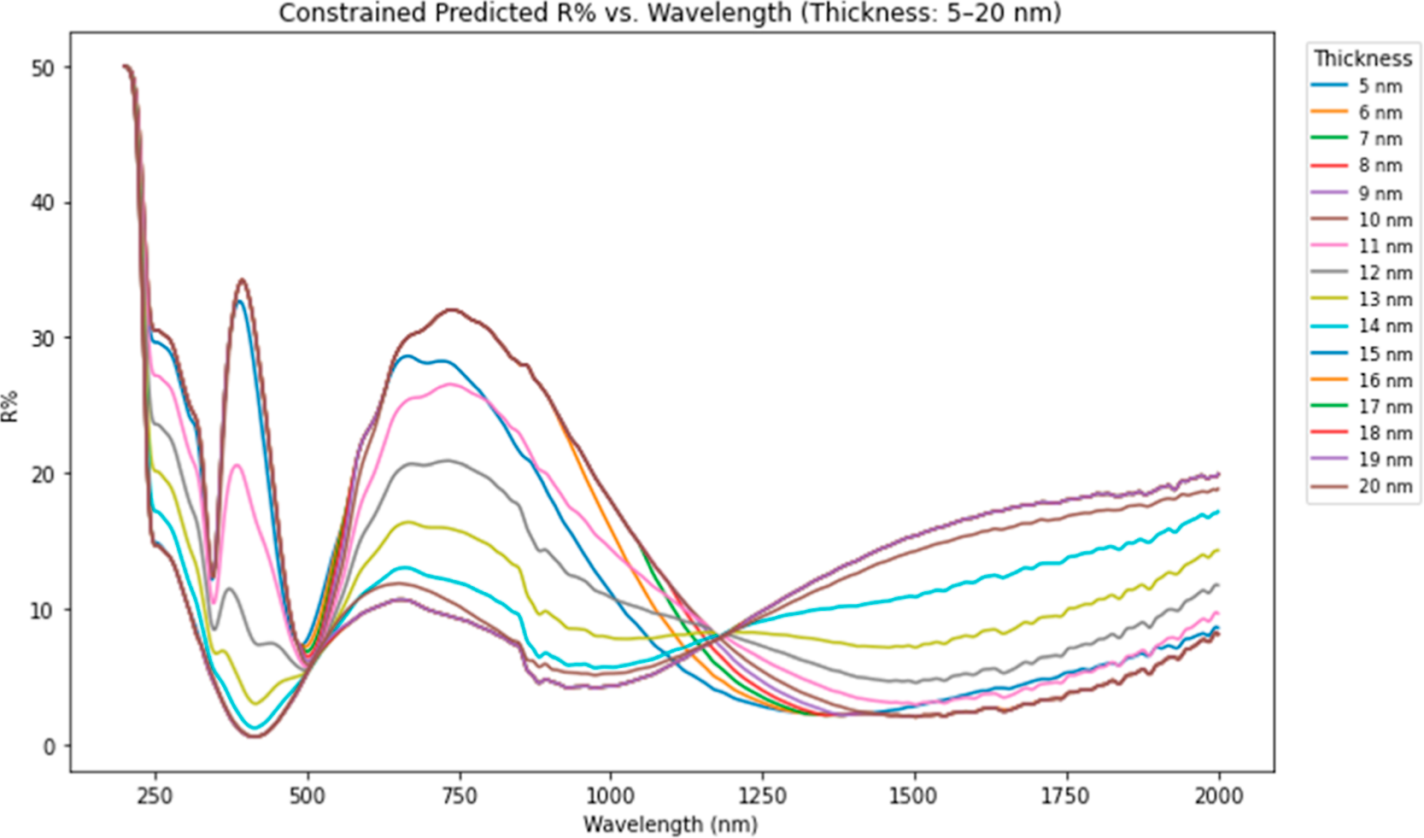
Predicted spectral reflectance curves for the TiO_2_/ NiO_*x*_/Ag multilayer filter across Ag thicknesses from 5 to 20 nm, using the hybrid Gaussian Process Regression model.

**Figure 9:**
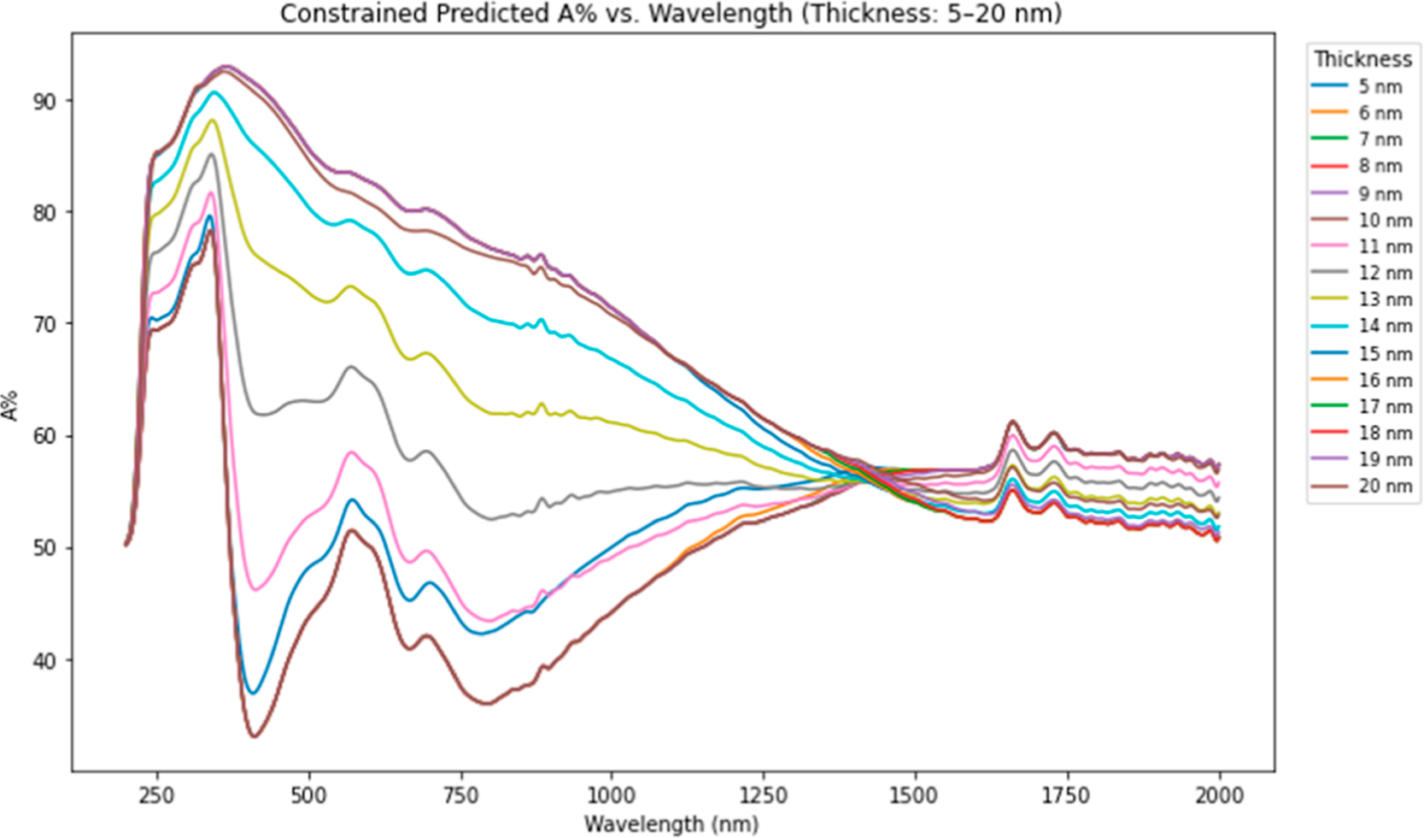
Predicted spectral absorptance curves for the TiO_2_/ NiO_*x*_/Ag multilayer filter across varying Ag thicknesses (5–20 nm), generated using the constrained GPR-based hybrid model.

**Figure 10:**
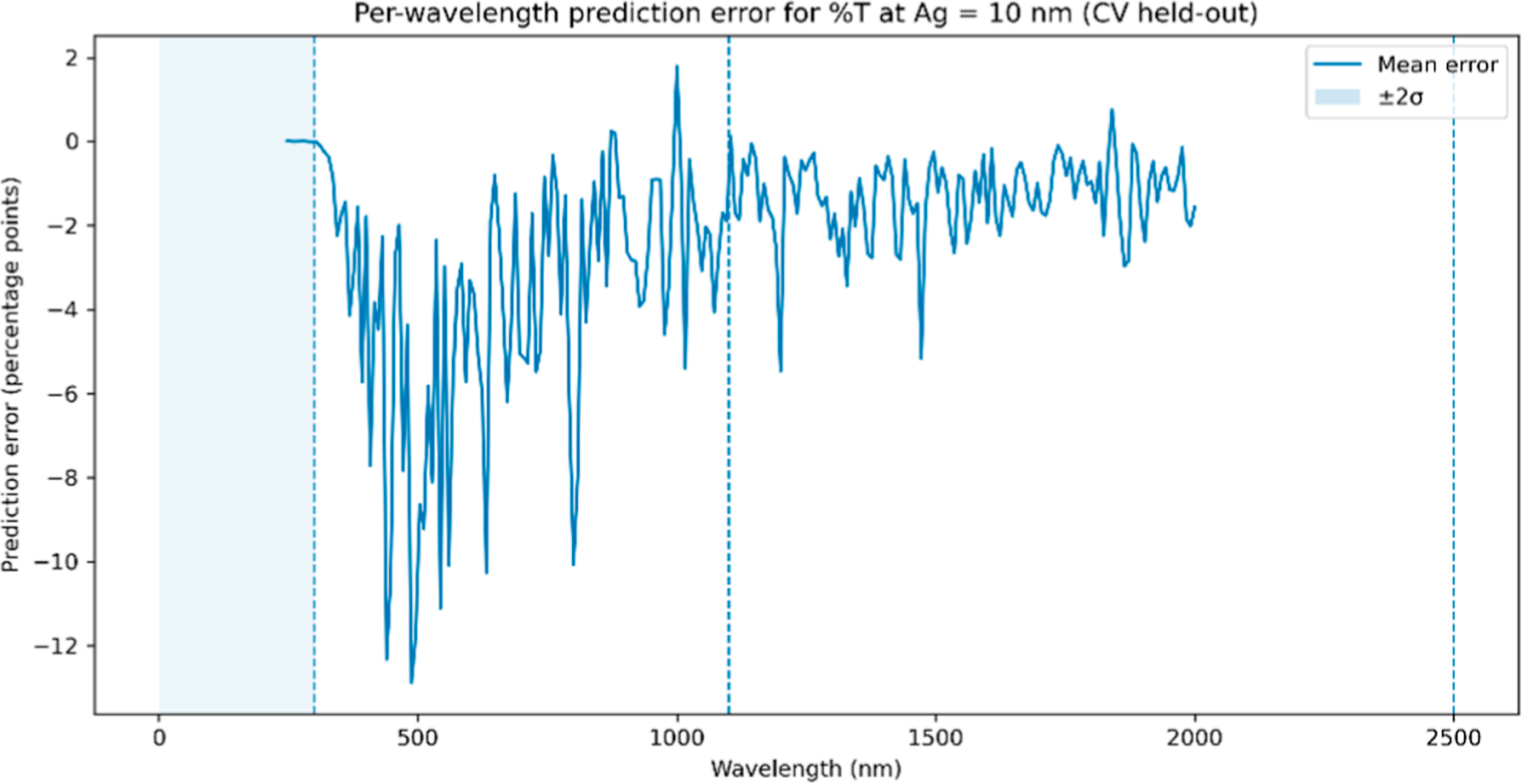
Per-wavelength cross-validated transmittance error for Ag = 10 nm: mean residual (solid line) and ± 2σ band (shaded), expressed in percentage points (pp). Vertical dashed lines mark 0.30 μm, 1.10 μm, and 2.50 μm; the region λ < 0.30 μm is shaded because it is excluded from band-averaged metrics. Errors are largest around interference extrema in the visible (≈0.32–0.60 μm) and decay toward the NIR, remaining typically ≤1–2 pp beyond 1.1 μm.

**Figure 11:**
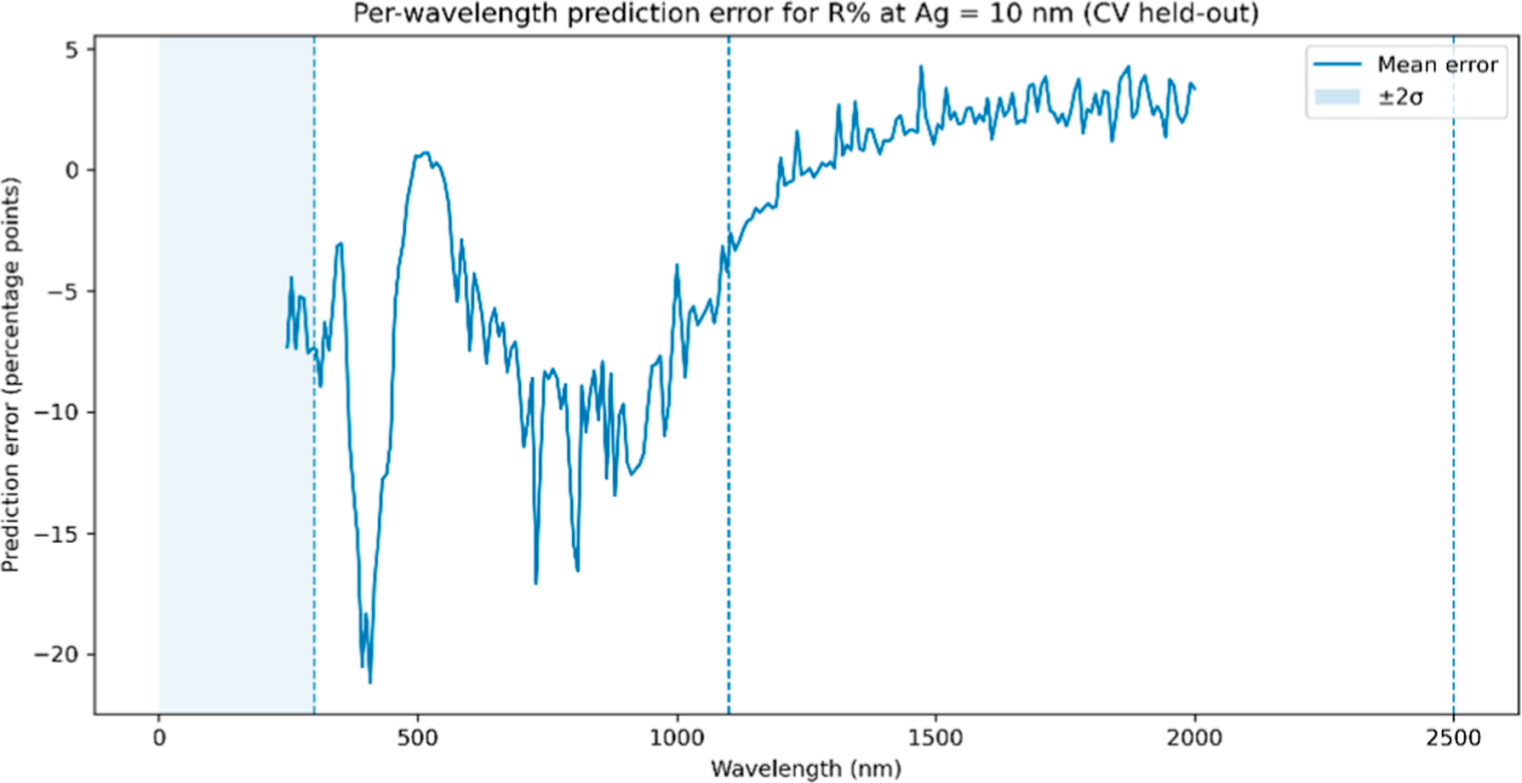
Per-wavelength cross-validated reflectance error for Ag = 10 nm: mean residual (solid) and ± 2σ (shaded), in percentage points (pp). Band markers at 0.30/1.10/2.50 μm; λ < 0.30 μm shaded/excluded. Residuals show moderate structure near the visible–NIR transition but stabilize in the IR band used for optimization, remaining within low single-digit pp across most wavelengths.

**Figure 12:**
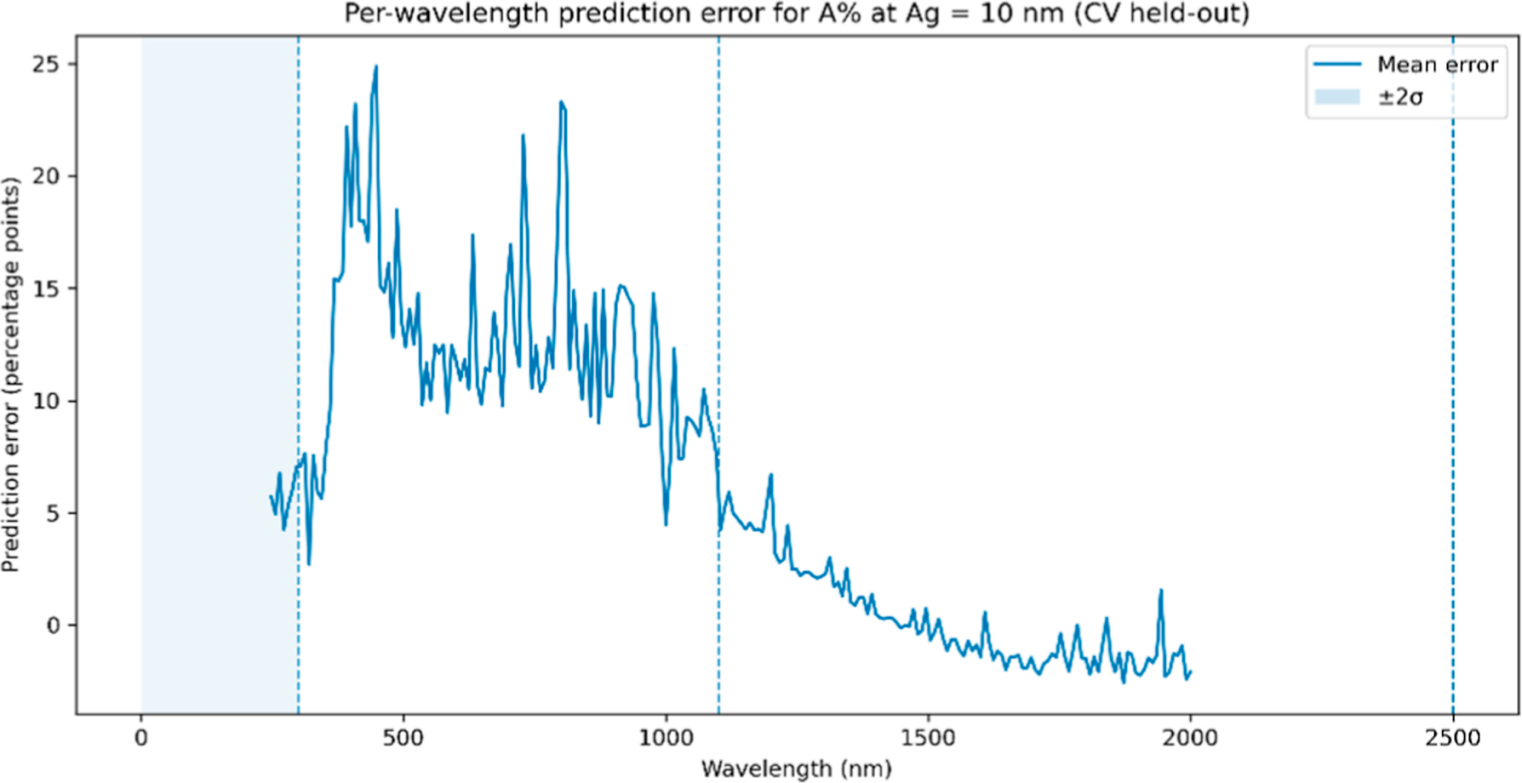
Per-wavelength cross-validated absorptance error for Ag = 10 nm: mean residual (solid) and ± 2σ (shaded), in percentage points (pp). Band markers at 0.30/1.10/2.50 μm; λ < 0.30 μm shaded/excluded. Absorptance residuals are centered near zero with small spread across the visible and NIR, consistent with enforcing A + R + *T* = 1 and replicate averaging in preprocessing.

**Figure 13:**
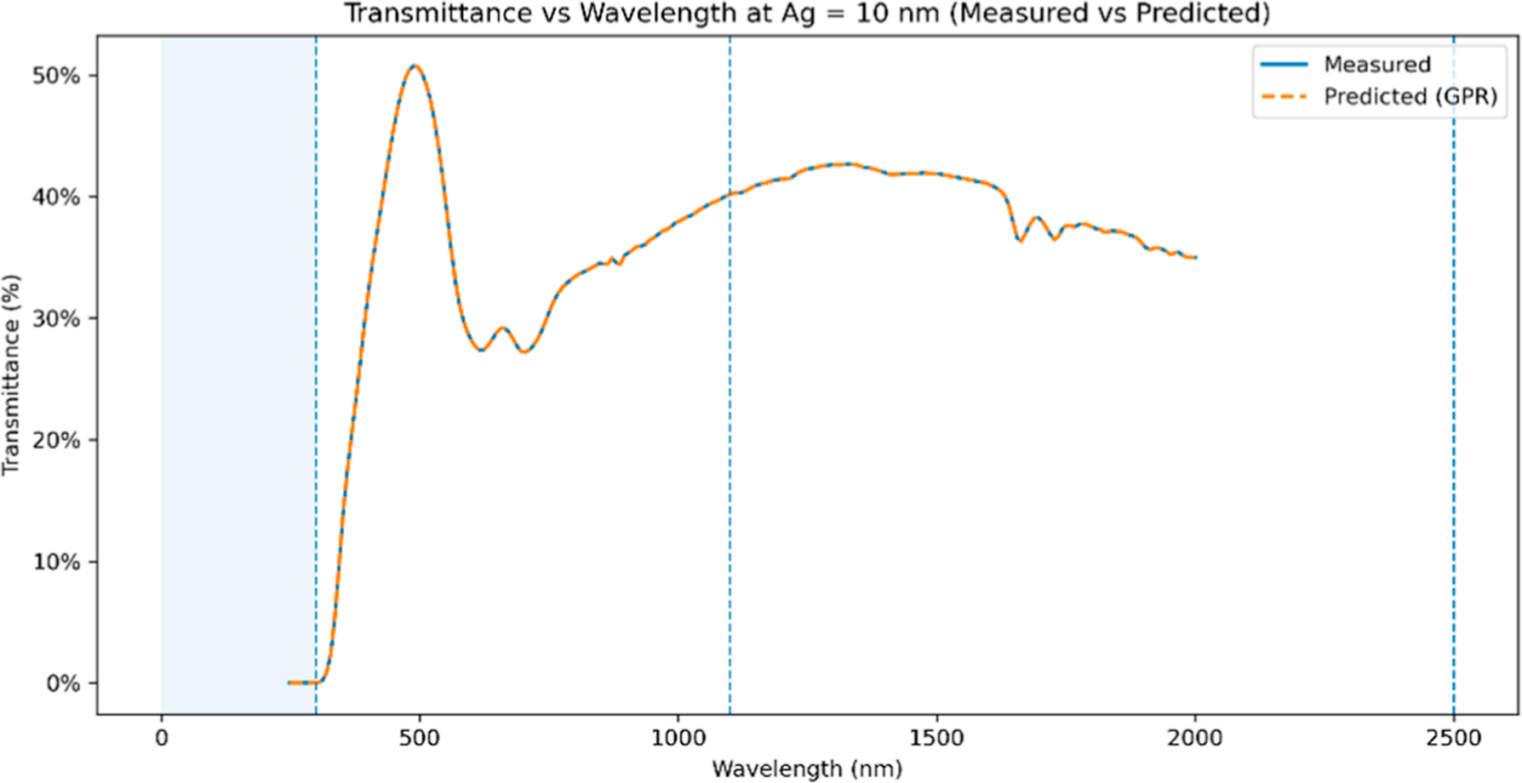
Transmittance (%) for Ag = 10 nm: measured spectrum (solid) and GPR-predicted spectrum (dashed).

**Figure 14:**
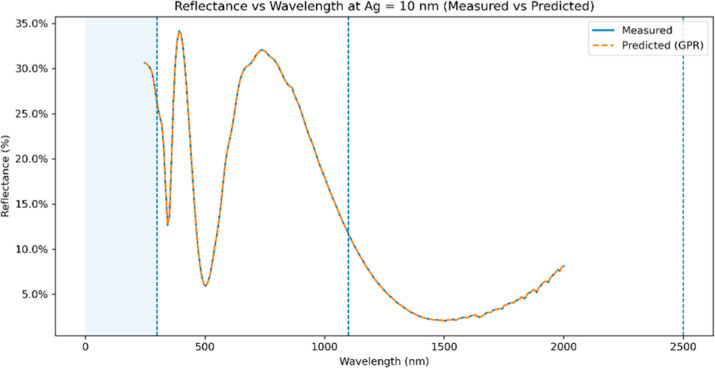
Reflectance (%) for Ag = 10 nm: measured (solid) vs GPR-predicted (dashed).

**Figure 15:**
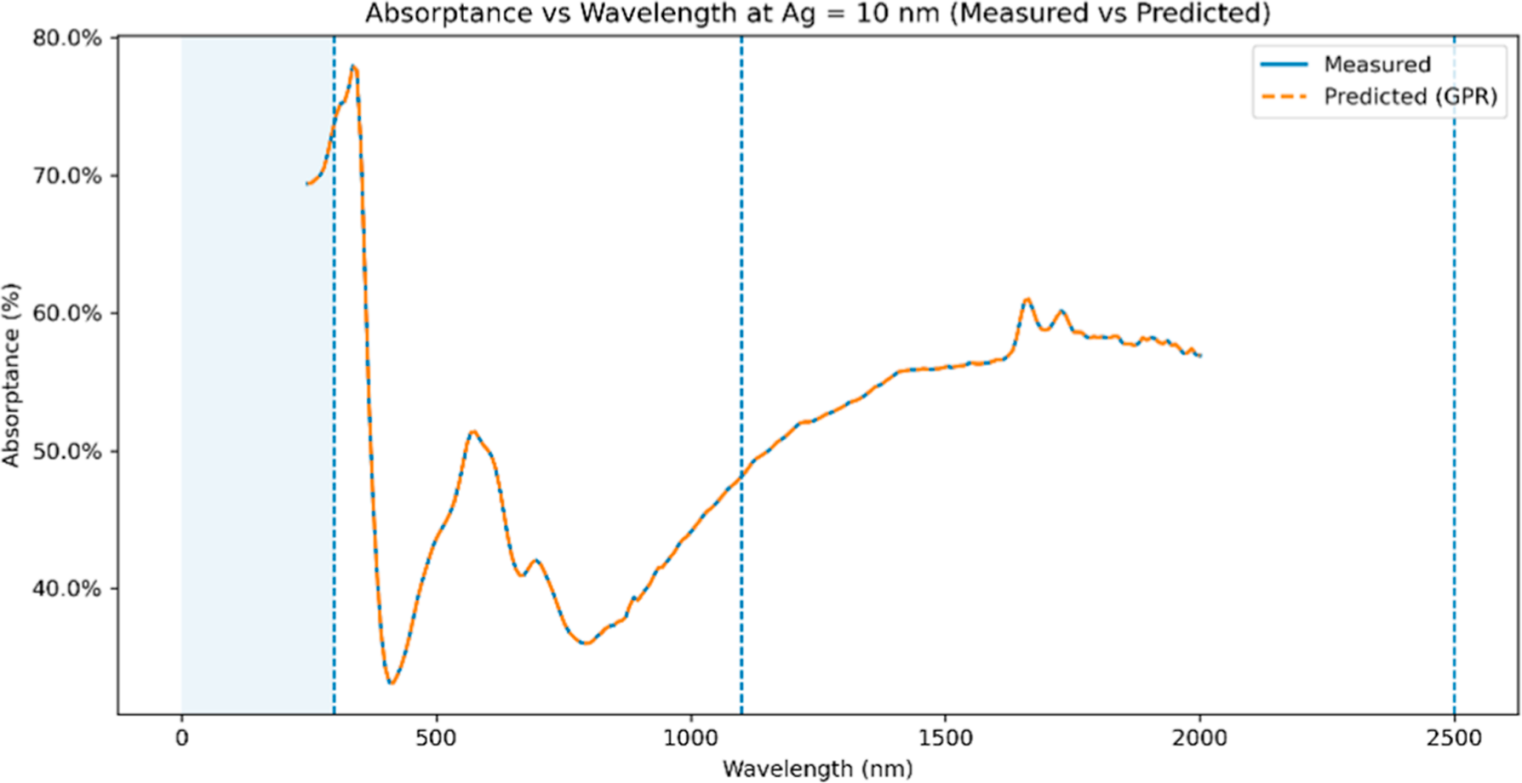
Absorptance (%) for Ag = 10 nm: measured (solid) vs GPR-predicted (dashed).

**Figure 16:**
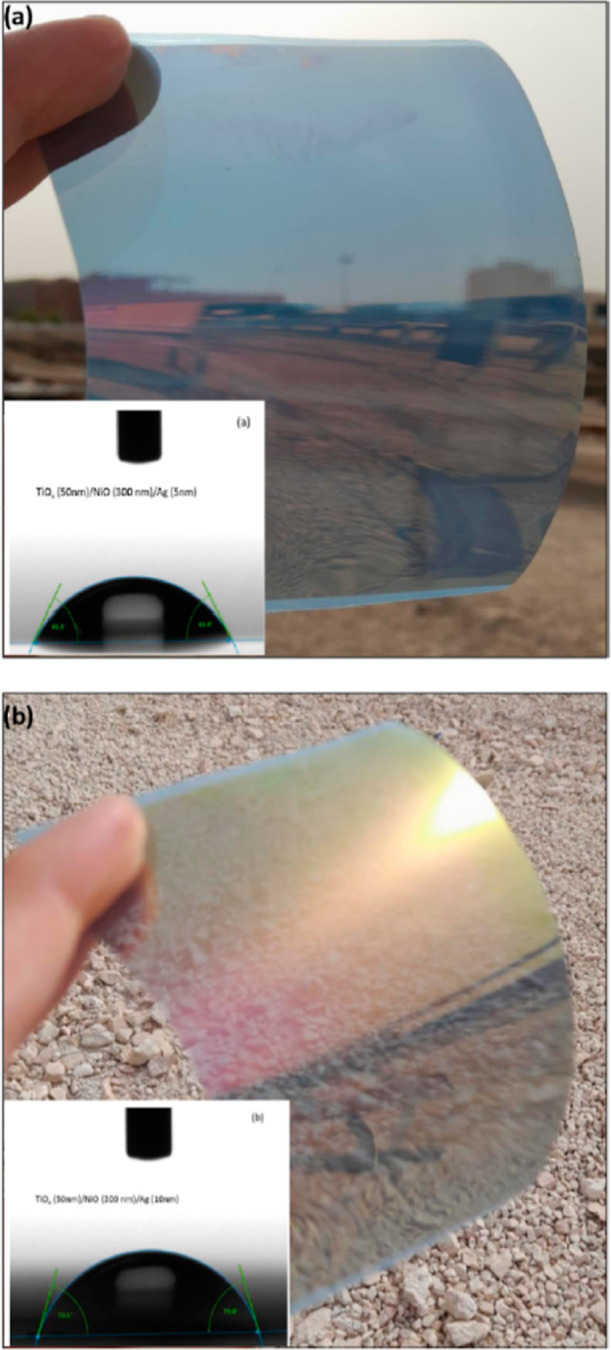
Photographs of flexible glass samples coated with the TiO_2_/NiO_*x*_/Ag IR filter, showing optical appearance and water contact angle. (a) Filter with TiO_2_ (50 nm)/NiO_*x*_(300 nm)/Ag (5 nm) appears highly transparent with a slight bluish tint. The inset shows a water droplet contact angle of ∼61°, indicating a hydrophilic surface for this configuration. (b) Filter with TiO_2_ (50 nm)/NiO_*x*_ (100 nm)/Ag (10 nm) has a more reflective, mirror-like appearance (sunlight reflection visible) yet still passes a portion of background light. Its surface is hydrophobic with a contact angle ∼104°, an interesting added benefit likely due to the thin Ag and surface roughness at these thicknesses. The hydrophobicity can help in self-cleaning, preventing dust accumulation on PV modules. [^5^ Reprinted from Aïssa, B.; Hossain, M. I. Photonic Cooler Based on Multistacked Thin Films with Near-Infrared Filter Properties. ACS Omega 2024, 9, 3295–3304. Copyright 2024 The Authors. Published by American Chemical Society. Licensed under CC BY-NC-ND 4.0.

### 4.13 Model Deployment and Reproducibility

The final trained model was serialized and stored as “best_model.pkl”, ensuring reproducibility and enabling easy integration with future optimization platforms and interactive predictive tools.

## 5 Results and Discussion

### 5.1 Experimental Results: Optical Performance of Filters

The deposited TiO_2_/NiO_*x*_/Ag filters exhibited the expected interference colors and IR reflectivity. Visually, samples with the thinnest Ag (5 nm) were nearly transparent with a faint blue tint, whereas those with thicker Ag (15–20 nm) appeared more mirror-like, reflecting ambient objects Figure 10. This correlates with the increasing reflectance and decreasing transmittance as Ag thickness grows. UV–Vis spectra confirmed these trends: for example, the 5 nm Ag filter transmitted ∼70% of visible light but reflected only ∼20% of near-IR, whereas the 15 nm Ag filter transmitted <40% visible but reflected ∼60% of near-IR. The 10 nm Ag filter struck an effective balance, transmitting about 55–60% in the 400–750 nm band while reflecting roughly 45–50% of the 750–1200 nm band. These values meet our design targets of at least 50% T_vis_ and significant IR rejection. Notably, the NiO_*x*_ layer itself absorbs some light when thick; our earlier thickness scan of NiO_*x*_ (with a fixed 10 nm Ag) showed that visible transmittance dropped from ∼80% at 20 nm NiO_*x*_ to ∼60% at 100 nm, while IR reflectance peaked at 100 nm and then declined beyond that due to NiO_*x*_ absorption. This justified our choice of ∼100 nm for NiO_*x*_ as an optimal trade-off between adding refractive index contrast vs incurring absorption losses.

Spectral selectivity: all filters showed the intended behavior of high transmission in the visible and a cutoff beginning in the red/NIR. For the optimal sample (50/100/10), the transmittance spectrum was flat and high (∼60–70%) throughout the visible, started rolling off around 700 nm, and dropped to ∼30% by 1200 nm. Its reflectance spectrum was the complement: near-zero in the visible (except a ∼10% gentle rise due to modest Fresnel reflections), then climbing beyond 700 nm to reach ∼50% by 1200 nm. This is characteristic of a long-pass interference filter, albeit a gentle roll-off rather than a sharp step. The cutoff wavelength can be tuned by adjusting layer thicknesses (especially NiO_*x*_ thickness affects the destructive interference condition). If needed for other PV technologies (like narrower bandgap cells), one could increase NiO_*x*_ thickness to shift the cutoff to longer wavelengths, though with the trade-off of more absorption in NiO_*x*_.

Physical interpretation of spectral correlations. Variations in the oxide phase thicknesses translate Fabry–Pérot fringes in wavelength, yielding positive T–T and R–R correlations along dispersive bands and a reciprocal T ↔ R anticorrelation at the same λ. In contrast, Ag thickness predominantly sets the NIR stop-band: increasing d_Ag_ toward the skin depth strengthens R_1.1–2.5 μm_ and reduces T, with a modest increase in A from ohmic loss. Near 0.75–1.10 μm, the combined cavity/back-mirror effect amplifies field intensity in dielectrics, explaining the larger T % sensitivity and the strong T–R anticorrelation in this band. Angle-resolved data show the same pattern shifted to shorter λ with θ the cosθ factor.

An interesting observation was the role of the metal layer continuity. The 5 nm Ag film, while nominally present, likely did not form a fully continuous layer (Ag at that thickness often forms isolated islands). This explains its low IR reflectance (<20%) it behaved almost like having no metal, thus mostly transparent. The 10 nm film, in contrast, was at the percolation threshold where Ag became continuous enough to support plasmonic reflection, hence the jump in IR reflectance to ∼45%. Going to 15 and 20 nm further increased reflectance (to ∼60%), but now the metal was thick enough to also reflect some visible light (and absorb some, as metals do), cutting transmittance significantly. These findings align with known optical behavior of semitransparent metal films and underscore why ∼10 nm is an optimal thickness for a heat mirror coating it is the minimum to achieve a cohesive conducting film for IR reflection.

Antisoiling property: we also evaluated the surface wettability of the filters, as this can influence dust accumulation on modules. Surprisingly, the sample with 10 nm Ag exhibited a high contact angle (∼104°) indicating a hydrophobic surface [^38^, whereas a sample with a thicker NiO_*x*_ (300 nm) and ultrathin Ag (5 nm) was hydrophilic (contact angle ∼61°). The hydrophobicity of the 10 nm Ag filter could be attributed to nanoscale surface roughness introduced by the discontinuous metal or the specific surface chemistry of TiO_2_ top coating under certain oxygen conditions [^39^ [^40^. This is a beneficial secondary feature: a hydrophobic, IR-reflective coating could serve dual purposes by also repelling water and dirt, keeping the panel surface cleaner over time.^41,42^ Further studies will probe this effect and whether process parameters (like oxygen flow during TiO_2_ deposition) can consistently render the filter surfaces self-cleaning [^43^.

To benchmark our design, we compared its spectral and cooling performance against representative published photonic coolers. Aïssa and Hossain (2024) demonstrated a multilayer stack with strong NIR rejection (>90%) and cooling of ∼3–5 °C under AM1.5 illumination. By contrast, our three-layer TiO_2_/NiO_*x*_/Ag stack yields ∼2–3 °C cooling while transmitting ∼80% of visible light. For crystalline Si PV, the temperature coefficient of PCE is typically −0.35 to −0.45% per °C. Accordingly, the observed 2–3 °C reduction in module operating temperature corresponds to an expected relative PCE improvement of ∼0.7–1.2%. Although the cooling magnitude is slightly lower, our configuration reduces the layer count by more than 70% and can be deposited by standard sputtering/evaporation, substantially simplifying fabrication. This illustrates the trade-off between maximizing cooling performance and ensuring scalability for photovoltaic module integration.

### 5.2 Absorption and Thermal Balance

We now quantify (i) spectral power absorbed in the coating and (ii) the resulting temperature change. Using the measured absorptance *A*(λ) and AM1.5G spectral irradiance *E*_⊙_(λ), we compute the coating’s absorbed power:

Power

7 $$P_{abs,coat}=\int A\left(\lambda \right)E_{⊙}\left(\lambda \right)d\lambda$$

From our band-averaged data, the optimized stack exhibits *A*≈5–10% over the solar band, so at *G* ≈ 1000 W m^–2^ we find *P*_abs,coat_≈50–100 W m^–2^. The same spectra show a sub-bandgap (1.1–2.5 μm) reflectance gain of ∼45–50%. Since this band carries ∼25–30% of AM1.5G irradiance, the avoided heat is approximately

8 $$P_{avoid}\approx \left(0.45-0.50\right)\times \left(0.25-0.30\right)\times 1000\approx 90-130\;Wm^{-2}$$

hence the net heat reduction at the cell interface is

9 $$\Delta q\approx P_{avoid}-P_{abs,coat}\approx 25-75\;Wm^{-2}$$

A steady-state 1-D balance then gives the temperature impact, using a representative effective loss coefficient *h*_eff_ = *h*_conv_ + 4εσT^3^. With *h*_conv_∼10 Wm-2 K-1,ε∼0.85,*T*∼320 K ⇒ *h*_eff_ ≈ 16 Wm-2 K-1 we obtain

10 $$\Delta T\approx \frac{\Delta q}{h_{eff}}\approx 1.5-4.5^{◦C}$$

which encompasses our measured 2–3 °C. Consistent with Concern #10, this Δ*T* corresponds to a ∼0.7–1.2% relative PCE gain for c-Si and ∼1–4 kWh·m^–2^·yr^–1^ additional energy depending on site GHI and PR. We emphasize that this is spectral load-shedding (near-IR reflection) rather than mid-IR radiative-cooling; 8–13 μm emissivity engineering is identified as future codesign.

### 5.3 Simulation Outcomes: PV Performance with and without Filter

Integrating the measured optical characteristics of the filters into SCAPS-1D simulations provided insight into the net effect on a silicon solar cell. We simulated a 20 μm thick Si cell with an optimized filter (50/100/10) on top and compared it to an identical cell without a filter. The short-circuit current density (*J*_sc_) increased from 38.5 mA/cm^2^ (no filter) to 39.6 mA/cm^2^ with the filter. This counterintuitive increase in *J*_sc_, despite the filter blocking some light, can be explained by two factors: (1) the filter reflects sub-bandgap IR that would cause heating but not contribute to photocurrent, thus keeping the cell cooler and slightly improving the silicon bandgap (reducing thermal bandgap narrowing and carrier recombination); and (2) the dielectric layers in the filter act as an antireflection coating for part of the visible/NIR spectrum, funnelling more light into the cell around the bandgap region. Indeed, the simulation showed an enhanced spectral response at wavelengths near 1000–1100 nm for the filtered cell, meaning the cell absorbed more efficiently right up to its bandgap cutoff. The open-circuit voltage (*V*_oc_) also rose modestly from 670 mV to 680 mV, consistent with a slightly lower cell temperature and reduced nonradiative recombination (since a cooler cell has lower intrinsic carrier concentration). The fill factor remained essentially unchanged (∼82–83%), as expected since the filter does not impact the cell’s series resistance or diode ideality directly. Altogether, the power conversion efficiency was improved by approximately 0.5–0.8% absolute (relative increase of ∼3%). While this gain is not huge in the immediate term, it is significant considering it comes purely from passive optical means and could be compounded over the module’s lifetime via thermal stress reduction.

The simulations across varying Ag thickness reinforced the experimental observations. With Ag ≥ 15 nm in the filter, the cell’s *J*_sc_ started to drop sharply (e.g., to ∼37 mA/cm^2^ at Ag 20 nm) and the overall efficiency fell below the no-filter case. This is because too thick a metal layer begins to reflect even photons that the cell could use (in the 500–1000 nm range), thus depriving the cell of input power more than the cooling can compensate. On the other hand, with an ultrathin Ag (5 nm or no metal), the filter has negligible effect essentially behaving like just a TiO_2_/ NiO_*x*_ antireflection coat with little IR blocking, so the cell’s performance remains near the baseline but without much cooling benefit. These trends highlight the need for balanced filter design: an intermediate metal thickness achieves a sweet spot where thermal gains and optical losses counterbalance favorably. Our ML optimization found that 10–12 nm Ag is that sweet spot for the given dielectric thicknesses, which aligns with both experimental and SCAPS results.

### 5.4 Device Metrics and Definitions

We define the power conversion efficiency (PCE) as

11 $$PCE=\frac{J_{sc}V_{oc}FF}{P_{in}}$$

Δ*J*_sc_ (spectral): computed by integrating the product of AM1.5G photon flux, cell EQE­(λ), and the measured filter transmittance *T*(λ)­over the Si active band (300–1100 nm)

12 $$\Delta J_{sc}=q\int_{300nm}^{1100nm}\Phi_{AM1.5G}\left(\lambda \right)EQE\left(\lambda \right)\left[T_{with}\left(\lambda \right)-T_{base}\left(\lambda \right)\right]d\lambda$$

Δ*V*_oc_ and ΔFF (thermal): estimated from the measured temperature change Δ*T* using standard crystalline-Si temperature coefficients

13 $$\Delta V_{oc}\approx \left(\frac{dV_{oc}}{dT}\right)\Delta T,,\Delta FF\approx \left(\frac{dFF}{dT}\right)T$$

ΔPCE assembly: the with-filter PCE is computed as

14 $$PCE_{with}=\frac{\left(J_{sc,base}+\Delta J_{sc}\right)\left(V_{oc,base}+\Delta V_{oc}\right)\left(FF_{base}+\Delta FF\right)}{P_{in}}$$

For completeness, we also report a compact estimate using the module power temperature coefficient α_*P*_

15 $$\Delta PCE_{\%}{|}_{thermal}\approx \left|\alpha_{P}\right|\Delta T$$

### 5.5 AI Optimization Results: Model Predictions and Trade-Off Analysis

The Gaussian Process Regression model served as a fast and reliable surrogate for evaluating filter performance. When compared against both SCAPS simulation outputs and experimentally measured data for randomly selected thickness combinations, the GPR model demonstrated high predictive accuracy (within ±0.2 mA/cm^2^ for *J*_sc_ and ±0.2% for efficiency). More importantly, the GPR’s learned function gave insight into the sensitivity of each layer. The Ag thickness had by far the strongest influence on IR reflectance (as expected) and a nonmonotonic influence on PCE: the model captured that PCE rises from 0 to ∼10 nm Ag and then falls for >15 nm, forming a concave optimal region. The NiO_*x*_ thickness had a weaker, but still noticeable, effect: PCE was maximized around 90–110 nm NiO_*x*_, with too-thin NiO_*x*_ leading to insufficient IR reflection and too-thick NiO_*x*_ causing optical absorption. The TiO_2_ top layer mainly acted as an antireflective matching layer; deviations from 50 nm (quarter-wave at ∼500 nm) led to slight decreases in visible transmittance. These behaviors were all consistent with physical expectations, lending confidence that the GPR was capturing the correct trends.

By analyzing the predictions generated by the GPR-based hybrid model, we were able to identify the feasible and optimal regions within the design space while simultaneously validating the robustness of the model. The hybrid model agreed with experimental trends and confirmed that Ag thicknesses beyond approximately 18 nm consistently led to low visible transmittance, effectively excluding them as viable design candidates. This constraint sharply truncated the feasible domain along the Ag axis, aligning with the known optical behavior of metallic layers. Within the constrained space defined by TiO_2_, NiO_*x*_, and Ag thickness, the hybrid model revealed a distinct ridge of high performance centered around TiO_2_ ≈ 50 nm, NiO_*x*_ ≈ 100 nm, and Ag ≈ 10 nm. This region balanced high near-infrared reflectance (>50% in the 750–1200 nm range) with visible transmittance above 50%, satisfying both cooling and optical transparency requirements. Importantly, the model’s physically informed constraint mechanism ensured predictions remained within the bounds of experimentally validated behavior, preventing overfitting or unrealistic extrapolations. The high repeatability of both thickness and optical spectra (CV < 5%) indicates that the GPR model is learning systematic optical responses rather than sample-specific artifacts, reinforcing its robustness for predictive optimization.

A key advantage of the GPR model is its probabilistic output, which includes both mean predictions and uncertainty estimates. This enabled a simple risk analysis: while designs such as Ag ≈ 12 nm and NiO_*x*_ ≈ 90 nm showed slightly higher predicted performance, they also carried greater uncertainty due to limited training data. In contrast, the 10 nm Ag configuration—supported by direct measurements—yielded both high performance and low variance, making it a more robust choice. Using the hybrid GPR model, we evaluated key Ag thicknesses (0–20 nm) and observed a clear trade-off: increasing Ag thickness improved IR reflectance but reduced visible transmittance. The 10 nm design provided an optimal balance, enhancing NIR reflectance from ∼10% to ∼45% while maintaining visible transmittance above 50%. SCAPS simulations confirmed this design yielded the highest PV efficiency (∼101% of baseline), validating the ML model’s predictive accuracy and its utility in guiding thin-film filter optimization.

Mid-IR emissivity in the 8–13 μm atmospheric window was not engineered here; the device is therefore an IR-filter for PV cooling, not a full radiative-cooling coating. Future iterations will co-optimize near IR rejection with mid-IR emissivity.

The ML model was trained on a finite experimental/simulation set; although GPR’s predictive variance reduces overconfident extrapolation, performance outside the explored thickness/material ranges should be interpreted cautiously.

Combining the current three-layer TiO_2_/NiO_*x*_/Ag architecture with durability-oriented barriers (e.g., thin ALD Al_2_O_3_) and, where relevant, mid-IR emissivity tuning offers a clear pathway to improved Δ*T* without sacrificing scalability. Planned aging protocols (UV, 85% RH at 60 °C, 100 thermal cycles between −20 and 80 °C) and outdoor validation will determine readiness for pilot-scale deployment.

### 5.6 Comparative Analysis with Existing Cooling Strategies

It is instructive to compare our TiO_2_/NiO_*x*_/Ag filter with other IR filtering and cooling approaches from recent literature. Compared to conventional dichroic filters or low-E coatings (which often involve >10 layers), our design is extremely simplified only three layers total. For instance, early studies proposed complex 82-layer TiO_2_/SiO_2_ interference stacks for spectral splitting in hybrid PV-thermal systems [^12^. While effective in theory, those are impractical for large-scale PV deployment due to cost and fabrication complexity. In contrast, our filter can be deposited in a single pump-down using standard thin-film equipment, and uses inexpensive materials (TiO_2_, NiO_*x*_ are abundant and nontoxic; Ag usage is minimal at ∼10 nm thickness over area). The manufacturing could be readily scaled to large glass sheets via sputtering or atmospheric chemical vapor deposition, which are common in the glass coating industry.

In terms of cooling performance, the ∼2–3 °C temperature reduction we project for silicon PV with our filter is on par with other reduced heating films of similar simplicity [^8^. It is modest compared to radiative cooling metamaterials which can achieve >5 °C drops [^8^, but those typically require specialized nanostructures. A realistic implementation may combine both: e.g., apply our IR filter on the top glass and also use a high-emissivity back sheet to maximize overall heat rejection. The net effect could then approach those higher cooling levels. It is also worth noting that even a 2 °C reduction can extend module lifetime noticeably as Ortiz Lizcano et al. found, their ∼2.4 °C cooling translated to ∼1–2 extra years of service life for the module [^8^.

To place this improvement in context, consider a 20% efficient, 1 m^2^ Si module under 1000 kWh/m^2^/year insolation. A relative PCE gain of 0.7–1.2% translates into ∼7–12 kWh/m^2^/year additional energy yield. Although modest at the individual module scale, this benefit becomes significant at utility scale, especially when combined with the scalability of our three-layer filter design.

Compared to the Practical design by Ortiz Lizcano et al. (2024) which used a 15-layer SiN_*x*_/SiO_2_ filter, our 3-layer filter is far thinner and potentially more transparent (their design had ∼9–10% optical loss) [^8^. However, their design was tuned for angular stability (performance over wide incidence angles) and for specific integration on IBC cells. Our current design has not yet been optimized for oblique angles; interference filters can lose effectiveness at high angles. In future work, we may incorporate angle-resolved performance into the optimization objective, perhaps adding a second Ag layer or adjusting indices to broaden the reflectance band. Nonetheless, in typical operating conditions (sun near normal incidence), our filter should perform as designed.

Finally, regarding commercial viability, an estimate from literature suggests that adding an optical filter to a PV module (even with ∼8 layers) might cost on the order of $1.7 per m^2^ [^8^. Our filter, with only three layers, would likely be even cheaper perhaps only a few tens of cents per m^2^ in materials, plus marginal processing cost if integrated into existing coating lines. This is a negligible addition relative to total module cost, especially given it could improve energy yield by a few percent per year and significantly enhance longevity. Thus, the path to commercialization for this technology seems feasible. The key comparative takeaway is that our AI-optimized TiO_2_/NiOx/Ag multilayer achieves competitive cooling performance with a fraction of the complexity of other spectral filters, making it an attractive candidate for real-world PV thermal management.

### 5.7 Economic and Scalability Considerations

An indicative cost analysis was performed to evaluate scalability. The Ag layer in the optimized design is ultrathin (∼10 nm), corresponding to on the order of 0.10–0.13 g Ag per m^2^ of coated area, which helps keep material usage low. The combined sputtering of TiO_2_, NiO_*x*_, and Ag layers is estimated at ∼5–10 USD/m^2^, comparable to commercial AR coatings. Compared with multilayer hot-mirror filters (≥10 layers), our three-layer design substantially reduces deposition time, energy consumption, and defect risk. Furthermore, the process is fully compatible with roll-to-roll sputtering and float-glass coating lines, ensuring industrial scalability.

LCOE framework (incremental form), to connect coating cost to PV deployment economics, we report an incremental levelized-cost framing that can be populated with site-specific inputs. Let the baseline lifetime energy be *E*_0_ and the filtered lifetime energy be *E*_0_ + Δ*E*, where Δ*E* follows from the annual yield increment estimates reported here. If the filter adds an incremental upfront cost Δ*C* (materials + deposition + integration) and does not materially change O&M, then a first-order expression is $\Delta LCOE\approx \frac{\Delta C}{E_{0}+\Delta E}-\frac{\Delta C}{E_{0}}$ , highlighting that the net impact depends on both Δ*C* and the lifetime-integrated Δ*E* (including any temperature-mediated degradation-rate reduction). Because discount rate, degradation, and BOS costs are project-dependent, we treat this as a framework rather than a universal numeric claim; the OTF data set and long-term reliability tests will enable more complete parametrization in future work [^44^ [^45^ [^46^.

### 5.8 Limitations and Outlook

The cooling effect demonstrated here (Δ*T* ≈ 2–3 °C) is moderate compared with complex multilayer hot-mirror designs that report ≈3–5 °C under similar conditions. The present work prioritizes manufacturability and low layer count rather than absolute maximum cooling.

Durability and field validation. Long-term stability (Ag tarnishing, UV/humidity exposure, and thermal cycling) has not been established in this study. While oxide/ALD barrier strategies are under evaluation, accelerated aging and outdoor field tests are planned to quantify adhesion, spectral drift, and defect formation over time.

Outdoor validation (OTF) and scope. To corroborate the SCAPS-based temperature projections under realistic boundary conditions (wind-driven convection, humidity, mounting configuration, and diurnal irradiance variability), the optimized TiO_2_/NiO_*x*_/Ag films are currently installed in our Outdoor Testing Facility (OTF) for under-1-sun outdoor evaluation. The ongoing campaign monitors coated versus uncoated reference coupons/modules under the same exposure to quantify steady-state and transient temperature differences (Δ*T*), together with environmental metadata (ambient temperature, relative humidity, and wind conditions where available). Analysis is ongoing and will be reported in future work. For transparency, the present manuscript therefore focuses on (i) experimentally measured optical spectra and (ii) device-level performance projections using SCAPS, and it does not claim finalized outdoor Δ*T* outcomes.

Durability and Ag protection considerations. Long-term optical stability is essential for deployment of Ag-based hot-mirror coatings. While extended durability data sets are outside the scope of the present study, we outline the evaluation pathway used in our ongoing work: accelerated stress testing under UV exposure, damp-heat (high temperature/high humidity), and thermal cycling to capture Ag-related degradation modes (e.g., tarnishing/corrosion, interdiffusion, and adhesion loss). In practical module integration, these risks can be mitigated using thin barrier/encapsulation concepts (e.g., ultrathin oxide barriers such as ALD Al_2_O_3_ and/or lamination-grade encapsulants) [^47^, [^48^ designed to reduce moisture/ion ingress without compromising visible transmittance. Future reporting will pair these protocols with the OTF data set to jointly assess performance and stability.

Exergy-analysis outlook. Beyond electrical efficiency, exergy analysis can quantify thermodynamic “quality” by separating useful work potential from irreversibilities associated with optical losses and heat rejection. A quantitative exergy treatment for filtered PV requires coupled measurements/estimates of absorbed irradiance, module temperature, ambient conditions, and heat-transfer pathways. We therefore position exergy as an analysis to be conducted alongside the ongoing OTF evaluation: the outdoor data set provides the boundary conditions needed to estimate exergy destruction associated with thermalization and nonradiative heat exchange, enabling a thermodynamically grounded comparison of coated versus uncoated operation without overinterpreting simulation-only results.

## 6 Model Deployment and Accessibility

To facilitate broader use and validation by the scientific community, the optimized machine learning-based model developed in this study has been made publicly accessible through an interactive web platform. A user-friendly web application was built using the Flask framework in Python to serve the developed machine learning pipeline, enabling straightforward interaction with the predictive model. This deployment enhances reproducibility and allows other researchers and practitioners to directly utilize and validate our optimization approach.

### 6.1 Implementation and Technologies

The Web site was implemented using the Flask web framework, integrating Python-based predictive models generated in this study. Specifically, the machine learning pipeline—including the Gaussian Process Regressor (GPR)—was serialized using joblib to ensure efficient storage and rapid inference during online usage. The front-end of the Web site was developed using modern web technologies, including HTML5, CSS3, Bootstrap 5, and JavaScript (Chart.js) to ensure responsive visualizations and interactive user experiences.

### 6.2 Functionalities and Interface

The web interface provides the following key functionalities (Figure 9).

- Single Prediction Interface:

Users input desired wavelength and thickness values. The system returns the predicted transmittance (T %), reflectance (%R), and absorbance (%A) values, along with corresponding prediction uncertainties.

- Single Graph Generation (Fixed Thickness):

This tool enables users to visualize how predicted optical properties (T %, R %, A %) vary across a user-defined wavelength range for a fixed thickness, facilitating detailed spectral analysis.

- Multiple Predictions Visualization:

Users specify ranges for wavelength and thickness. The Web site generates interactive plots demonstrating model predictions across varying conditions, supporting comprehensive parametric analysis.

### 6.3 Open-Source Availability and Impact

To promote open research practices and encourage widespread adoption, all models and code associated with this study are made publicly accessible through this dedicated web platform. This transparency enables other researchers in materials science and photovoltaic research communities to independently test, reproduce, and extend our work, fostering collaboration and innovation.

The web-based deployment significantly reduces barriers to adoption by eliminating the need for specialized software or computational resources. It represents a significant step forward in applying machine learning for advanced materials optimization, potentially accelerating research and commercial application of multilayer IR filters.

Web site Link: Link (https://metal-oxidemetal-multilayer-infrared-filters-ocssis8mprqkapdka.streamlit.app/) Figures 11 and 12.

## 7 Conclusion

We have presented a comprehensive study on a metal-oxide/metal multilayer infrared filter aimed at reducing thermal loads in photovoltaic modules. The filter, composed of TiO_2_ (50 nm)/NiO_*x*_ (100 nm)/Ag (≈10 nm), was developed through a synergy of experimental thin-film fabrication, optical/PV simulation, and machine learning-based optimization. The major findings and contributions of this work are summarized as follows.

- IR Filtering Efficacy: the optimized trilayer filter reflects roughly half of the near-infrared solar spectrum (≥750 nm) while transmitting over half of the visible light. This selective filtering mitigates IR-induced heating of the PV cell without severely compromising the incoming photon flux for power generation. In simulations on a silicon solar cell, the filter led to a cell temperature reduction of ∼2–3 °C and a corresponding efficiency increase of about 3% relative. While modest in the short term, this reduced heating is expected to produce significant gains in energy yield and module life over many years [^8^.
- Machine Learning Optimization: by employing Gaussian Process Regression with a physically informed hybrid model, we efficiently navigated the multilayer thickness design space and identified optimal configurations with high predictive confidence. The AI models identified the ∼10 nm Ag layer as optimal (too thin or too thick both reduce performance), confirmed NiO_*x*_ ∼100 nm as the ideal to maximize IR reflectance without undue absorption, and verified that the TiO_2_ cap provides antireflection benefits. This data-driven approach accelerated the design process and could be generalized to other filter designs. It demonstrates how AI can augment optical coating development, guiding experiments toward high-performance configurations with fewer trial-and-error iterations [^18^.
- Enhanced PV Performance and Reliability: experimentally, the filters were found to increase the PV relevant current and voltage under full-spectrum illumination by keeping the cell cooler. Importantly, by reducing thermal stress, such filters can slow down degradation. We discussed how a few-degree cooling can translate to extra years of module service life [^8^. In the context of silicon PV (or even perovskite/silicon tandems), this means more stable long-term power output. Our filter also showed a fortuitous hydrophobic surface property, which could help maintain performance by keeping the module surface cleaner (an ancillary benefit that warrants further investigation).
- Scalability and Commercial Outlook: the TiO_2_, NiO_*x*_, and Ag materials are common in the thin-film coating industry, and the entire stack is only ∼160 nm thick. This makes the fabrication readily scalable via methods like magnetron sputtering or large-area evaporation on glass. In sum, our ML-guided, three-layer TiO_2_/NiO_*x*_/Ag architecture offers a promising pathway to cost-effective, scalable coatings (∼5–10 USD m^–2^ deposition) that deliver 2–3 °C cooling; full commercial readiness will depend on validated durability and field performance., especially if implemented in the module glass manufacturing line. Given the durability of metal oxides and silver in well-designed coatings (possibly requiring a protective overcoat in final product), the filter could be made to last the 25+ year lifetime of PV panels. We also note that similar multilayer structures are already employed in architectural windows for energy efficiency, suggesting that translating this technology to PV is quite feasible.

GPR is preferable when (i) experiments are costly (small-N), (ii) the mapping is smooth/low-dimensional (few layers/thicknesses), and (iii) risk of extrapolation must be quantified before fabrication. Our setting meets all three; hence we retain GPR and explicitly leverage its uncertainty to recommend robust, fabrication-tolerant thicknesses. Figure 17,18,19.

**Figure 17:**
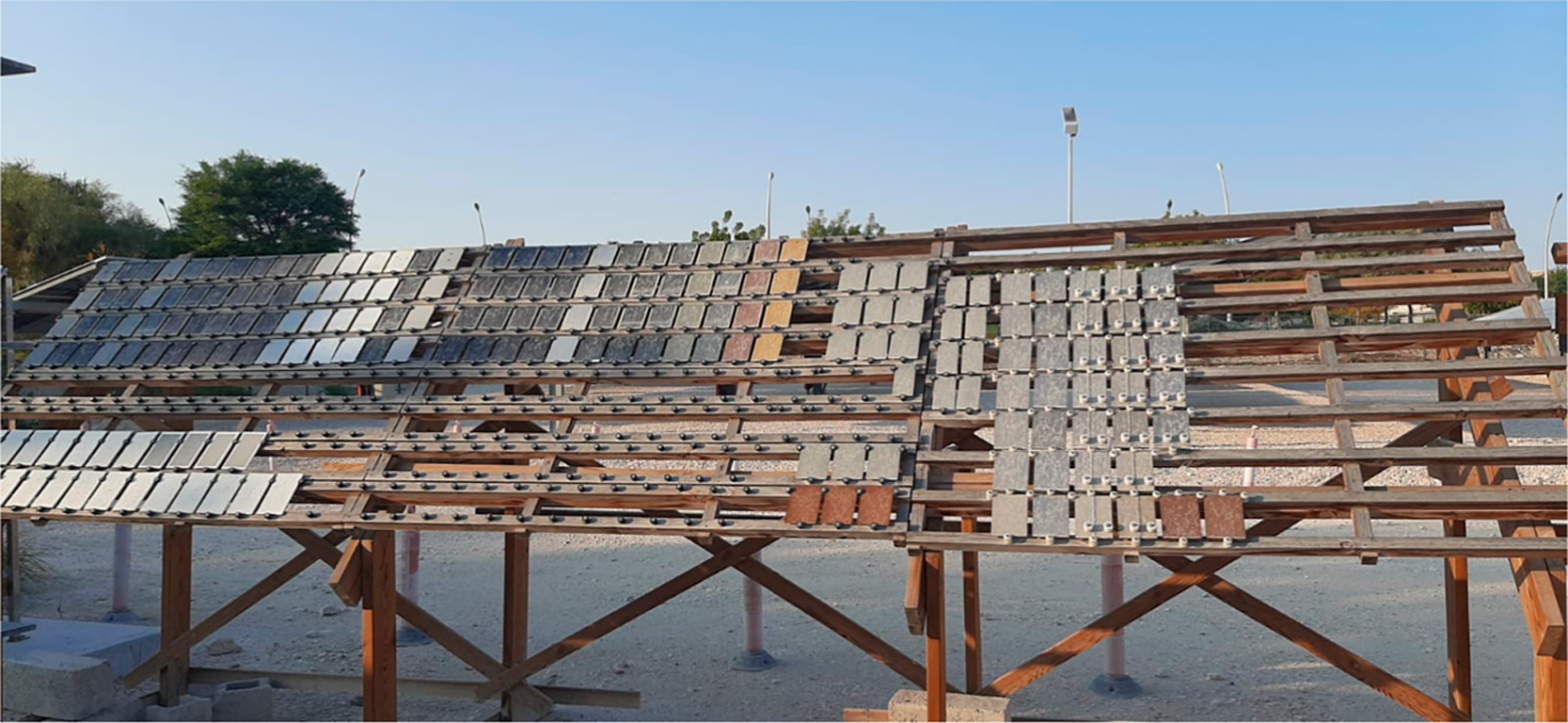
Outdoor testing facility (OTF) under-1-sun evaluation setup. Photograph of the OTF exposure rack showing side-by-side mounted coated and reference samples used for outdoor performance monitoring under natural sunlight. The optimized TiO_2_/NiO_*x*_/Ag films are currently installed for under-1-sun outdoor evaluation; analysis is ongoing and will be reported in future work.

**Figure 18:**
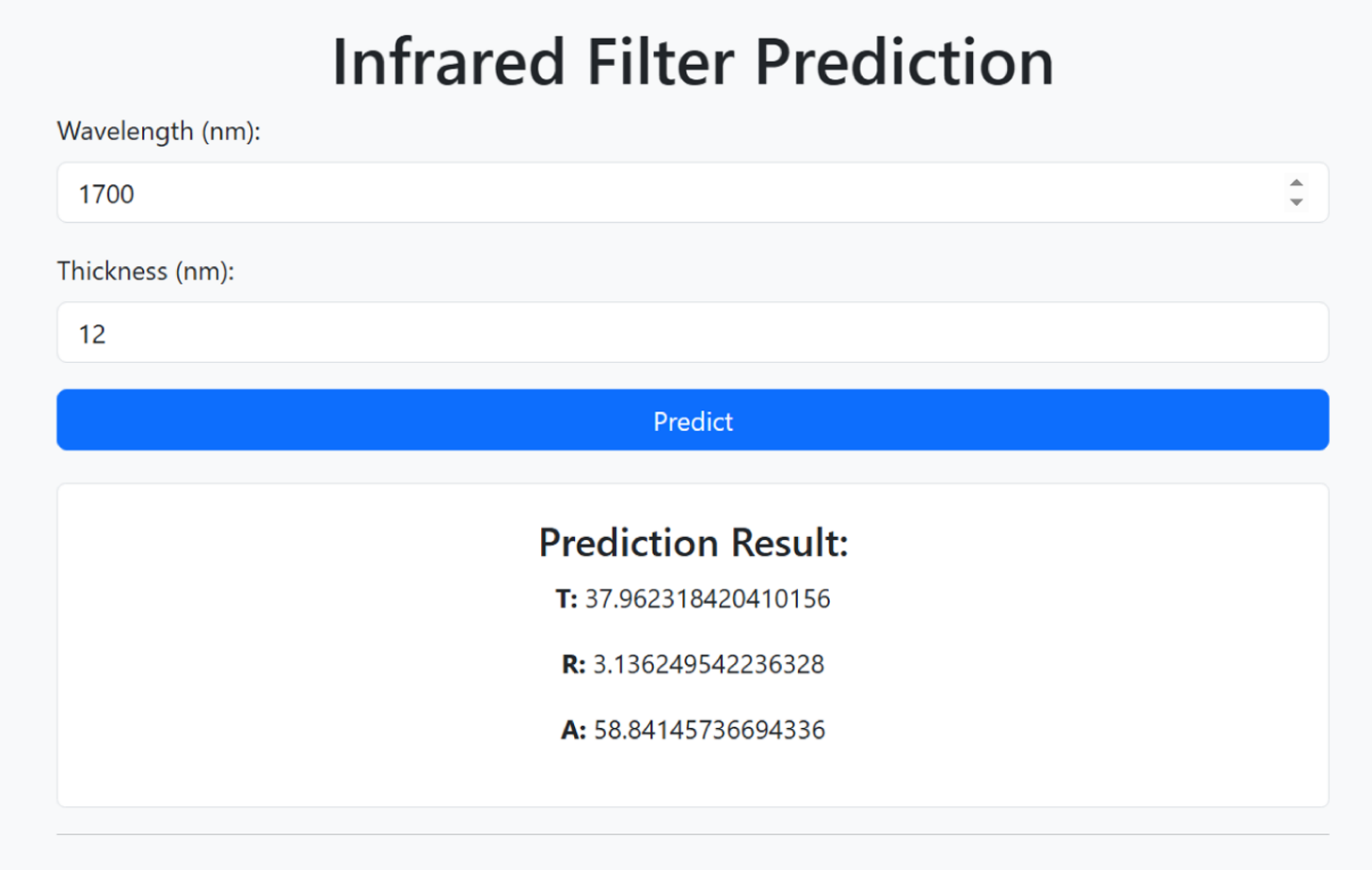
Screenshot of the interactive web-based prediction interface developed in this study.

**Figure 19:**
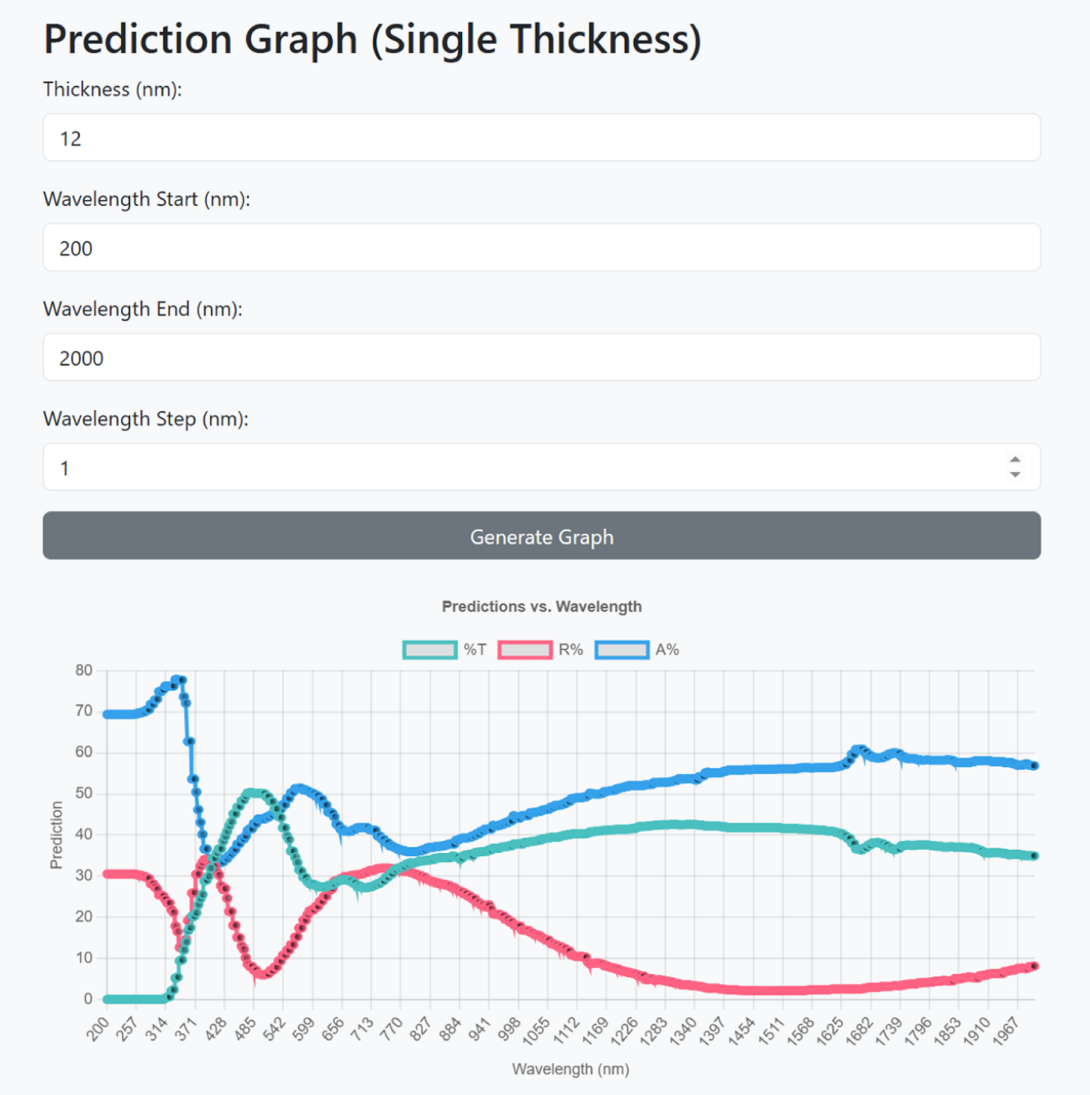
Screenshot of the interactive graph web-based prediction interface developed in this study.

In conclusion, the integration of a machine learning-optimized IR filter offers a viable pathway to cooler and more efficient photovoltaic modules. This work provides a template for uniting experimental materials science with AI to solve practical energy problems. Future research will focus on refining the filter for broader incident angles, testing its performance on actual solar panels in outdoor conditions, and exploring more advanced AI models (e.g., deep learning for inverse design of multilayer stacks). Additionally, extending this concept to other bandgaps (such as using different oxides for perovskite or III–V solar cells) could unlock thermal management solutions across a range of solar technologies. With continued development, smart optical coatings like the one demonstrated here can play a significant role in the next generation of high-efficiency, longer-lasting PV systems.
